# Using Less Processed Food to Mimic a Standard American Diet Does Not Improve Nutrient Value and May Result in a Shorter Shelf Life at a Higher Financial Cost

**DOI:** 10.1016/j.cdnut.2024.104471

**Published:** 2024-10-02

**Authors:** Julie M Hess, Madeline E Comeau, Angela J Scheett, Anne Bodensteiner, Allen S Levine

**Affiliations:** 1 United States Department of Agriculture, Agriculture Research Services, Grand Forks Human Nutrition Research Center, Grand Forks, ND, United States; 2 University of North Dakota, Grand Forks, ND, United States; 3 Department of Nutrition and Dietetics, University of North Dakota, Grand Forks, ND, United States; 4 Department of Food Science and Nutrition, University of Minnesota, Minneapolis, MN, United States

**Keywords:** clean eating, ultra-processed, Standard American Diet, whole food, processed food

## Abstract

**Background:**

The “clean eating” trend suggests that consuming fewer processed foods is important for healthy diets. Yet, a diet of mostly ultra-processed foods (UPFs) can meet recommendations from the Dietary Guidelines for Americans. Whether a diet comprised mostly of simple ingredient foods can provide a low-quality diet remains unexplored.

**Objectives:**

The objective of this study was to compare the diet quality, shelf stability, and cost of 2 similar nutrient-poor menus, one containing primarily UPFs and the other containing less-processed foods (LPW), as defined by the Nova classification system.

**Methods:**

A “Western” menu using LPW was developed to match the meals and recipes of a menu that contained more-processed foods (MPW). Processing level was determined using the Nova classification system. Final menus were assessed for nutrient quality and Healthy Eating Index (HEI) score. Shelf stability of foods/ingredients on both menus was determined from food storage guidance manuals. The condition of each food item when purchased (room temperature, frozen, refrigerated) was used to estimate the number of days until expiration. Food costs were determined from prices at grocery chains in Fall 2023.

**Results:**

The LPW had similar nutrient density and diet quality scores to the MPW (HEI scores of 44 and 43, respectively). The LPW included 20% energy (kcal) from UPFs, whereas the MPW included 67% energy from UPFs. Relative percentages of shelf-stable, frozen, and refrigerated foods were similar. Using the Kaplan-Meier survival analysis method, median time to expiration of the LPW menu items was 35 d compared with 120 d for the MPW items. The “per person” cost reflecting only the amount of the food used in the menu was $15.91/d for the LPW and $9.85/d for the MPW.

**Conclusions:**

Less-processed menus can have comparable diet quality with more-processed menus although being more costly and less shelf stable.

## Introduction

Both “ultra-processed” and “clean label” are popular topics in the fields of nutrition and food science [1,2]. However, neither descriptor has a clear or easy to implement definition, which has been the subject of much concern and controversy among the scientific community [[3], [4], [5], [6]]. Despite the lack of clear definitions, these terms have been adopted by consumers. According to consumer insights research conducted by the International Food Information Council, most Americans pay attention to the ingredient list on foods and beverages and take care to choose items with “clean” ingredients [7]. These “clean eaters” further describe their eating habits as consuming foods that are not highly processed, eating foods from the produce section, and eating organic foods and foods with a simple ingredient list [7]. The primary reason consumers provide for wanting to purchase clean label foods and beverages is a perception that these products are healthier than products that have “chemical-sounding” or “unfamiliar” ingredients, which consumers perceive as having potentially harmful effects [7]. This avoidance of chemical, unfamiliar, and artificial ingredients dovetails with consumer preference for natural flavors, preservatives, sweeteners, and colors [7].

Nutrition recommendations in some countries now use the term “ultra-processed” to describe foods with these types of “unfamiliar” ingredients. Dietary guidelines in Brazil [8], for instance, recommend consuming mainly unprocessed or minimally processed foods [9]. These guidelines recommend that consumers identify “ultra-processed” foods (UPFs) to avoid by looking at the ingredient list of foods and beverages, noting that products with 5 or more ingredients, ≥1 of which is an ingredient with an unfamiliar name not used in home culinary preparations, are likely to be UPFs. This definition of UPFs overlaps with the Nova classification system, which groups foods into 1 of 4 categories: *1*) unprocessed foods; *2*) processed culinary ingredients; *3*) processed foods; and *4*) UPFs [[10], [11], [12], [13]].

In short, there is a narrative among consumers and in some global dietary advice that “clean eating,” sometimes described as choosing less-processed foods with fewer ingredients, will improve diet quality and, therefore, health outcomes [14,15]. This message oversimplifies the scientific background about healthy dietary patterns and nutrient-dense foods while complicating the process of choosing foods and beverages to consume [16]. There is also no regulatory guidance at this time for use of the term “clean” on food packaging in the United States [5]. However, the 2025–2030 Dietary Guidelines for Americans (DGA) Scientific Advisory Committee is currently reviewing evidence about UPFs and associations with growth, body composition, and risk of obesity for consideration in future dietary guidance [17].

The average American diet would benefit from nutritional improvements. Americans tend to under-consume whole grains, fruits, vegetables, and dairy foods (which includes fortified soy milk and soy yogurt), relative to recommendations in United States federal dietary guidance, the DGA. The 2020–2025 DGA considers these food groups essential components of healthy dietary patterns. There are several options in grocery stores for foods from these groups that would be considered UPFs by the definitions in Brazil’s dietary guidelines and by the Nova classification system [18]. However, several whole-grain foods like packaged whole-wheat bread and whole-wheat tortillas as well as fruits and vegetables (some brands of raisins, canned greens, salsas, and dried fruits) and dairy foods (fortified soymilk, ultrafiltered milk) are considered UPFs according to the Nova classification system.

Yet, whole-grain bread, dried fruits, salsa, and dairy foods are examples of nutrient-dense UPFs that are resource-intensive (work, time, and money) to produce at home from fresh ingredients. Packaged foods can make nutrient-dense choices more accessible as they do not require the same investment of resources—both cost and labor—as homemade versions [19]. More-processed foods are often found to be less costly than their unprocessed or minimally processed alternatives [[20], [21], [22]]. To our knowledge, no study to date has examined the cost of more- and less-processed diets matched for food type and diet quality.

Processed foods may also increase accessibility to nutrient-dense foods by having a longer shelf-life and, therefore, being less likely to spoil relative to less-processed foods. Yet, although extending shelf-life has been discussed as a potential benefit of processing [23], to our knowledge, the potential difference in shelf-life of more- and less-processed dietary patterns has not previously been explored quantitatively by the nutrition science community.

Although packaged and processed food can be nutrient dense [18], the inverse of this statement can also be true: less-processed and “clean label” foods can be nutrient poor [24]. For instance, many foods prepared at home may be less processed but are not nutrient dense. Homemade cookies and other desserts, homemade pasta dishes or soups that are cream based, homemade lemonade and other sugar-sweetened beverages, and even items like homemade French fries, chips, and other fried foods are not necessarily nutrient rich. Yet, if prepared at home from “scratch” ingredients, these foods are considered less processed than premade alternatives. Previous research has indicated that recipes using less-processed foods can be as nutrient poor as recipes using more-processed alternatives [24]. An Australian study compared the nutrient content of recipes from “clean eating” blogs with control recipes with no clean eating claims and found that the “clean eating” blog recipes contained more protein, fiber, and fat than the control recipes but did not differ in terms of energy, carbohydrate, sugar, or sodium content [24]. Although the “clean eating” blogs promoted consuming more unprocessed foods, the actual nutrient content of “clean” breakfasts, snacks, smoothies, desserts, and treats did not differ from alternatives made with more-processed options [24].

The overall low quality of the American diet as indicated by Healthy Eating Index-2015 (HEI-2015) scores indicates that, across the United States population, most dietary patterns, regardless of processing level, are nutrient poor. The average HEI-2015 score for Americans ages 2 and older is 59 out of 100. The well-known low quality of American diets has led to the popularization of the abbreviation “SAD” as shorthand for “Standard American Diet” [25].

Given the discourse about the benefits of a “clean eating” approach to diet and its health benefits, the objective of this study was to assess whether a “SAD” or “Western” menu primarily comprised of less-processed foods (categories 1 and 2 of Nova, for example, unprocessed foods and processed culinary ingredients) would have a higher diet quality score or nutrient content compared with a similar Western menu primarily comprised of more-processed foods. That is, to compare the diet quality score between a less-processed Western menu (LPW) and a more-processed Western menu (MPW). This study also compares the cost of LPW and MPW menus and the relative shelf stability of foods used to prepare the LPW and MPW.

## Methods

### MPW and LPW development

A 2000-kcal Western-style menu was developed by research dietitians at the USDA-Agricultural Research Service Grand Forks Human Nutrition Research Center (GFHNRC) for use in feeding studies. This menu was used as the MPW. The LPW was developed for this study to match the meals and recipes in the MPW using less-processed ingredients. Both menus were developed using foods and nutrients identified as commonly consumed in quintile 1 of diet quality (HEI-2015) from the NHANES’s dietary assessment component, the What We Eat in America (WWEIA) survey (2011–2012). WWEIA represents eating patterns of a large, nationally representative sample of Americans. Quintile 1 was selected because consuming the lowest diet quality is associated with greater weight gain over time compared with higher diet quality [26]. Yet, because Americans tend to over-report “healthy” food items and under-report “unhealthy” foods, leading to potential over-inflation of diet quality [27], quintile 1 may be a better representation of average diet quality and eating patterns. Eating patterns in quintile 1 of diet quality are generally low in dark-green and orange and red vegetables as well as beans and seafood but high in energy from saturated fats and added sugars. The MPW can be found in Supplemental Table 1.

The HEI-2015 is a diet quality measure specifically designed to evaluate concordance with DGA recommendations for 9 adequacy components (total fruits, whole fruits, total vegetables, greens and beans, whole grains, dairy, total protein foods, seafood and plant proteins, and fatty acids) and 4 moderation components (refined grains, sodium, added sugars, and saturated fats). The HEI-2015 has a maximum score of 100. The HEI-2015 score of the MPW is 43 (out of 100), so the goal HEI-2015 score for the LPW was 43.

To create the LPW, less-processed versions of the foods on the MPW were identified from grocery retailer websites based upon their ingredient lists. For instance, the day 3 lunch on the MPW includes chili, cornbread with honey, sunflower seeds, chocolate milk, and a cookie. To generate a less-processed version of this meal, the chocolate milk was replaced with white milk, the canned beans in the chili recipe were replaced with dry beans prepared in a slow cooker, the cashew shortbread cookie was replaced with a less-processed Mexican wedding cookie, and the cornbread mix was replaced with a less-processed cornbread mix. A comparison of the ingredients in the cornbread mix on the MPW compared with the one from the LPW is provided below as an example:

Cornbread mix ingredient in original MPW: wheat flour, degermed yellow corn meal, sugar, lard (hydrogenated lard, butylated hydroxytoluene, and citric acid preservatives), contains <2% of baking soda, tricalcium phosphate, sodium acid pyrophosphate, monocalcium phosphate, salt, niacin, reduced iron, thiamin mononitrate, riboflavin, folic acid, wheat starch.

Cornbread mix ingredients in LPW: whole-grain cornmeal, whole-wheat flour, sugar, buttermilk powder, salt, baking powder (sodium acid pyrophosphate, sodium bicarbonate, cornstarch, monocalcium phosphate), baking soda.

Some items such as breads, stuffing, casseroles, jams and jellies, among others, required finding recipes, because packaged breads, stuffing, premade casseroles, and many commercial jams and jellies are Nova category 4 (UPFs) [28]. For these foods, recipes were selected based on the top Google search result for a recipe that did not utilize ultra-processed ingredients. For instance, to find a recipe for a less-processed jam without added pectin [29], the phrase “jam without pectin” was entered into a Google search, and the first option in the search results (“How to Make Basic Fruit Jam Without Pectin”) was used as the recipe for the jam in the LPW [30].

#### Nova classification determination

To verify Nova classifications of foods, recipes, and/or their constituent ingredients, all food items and a list of their ingredients were sent to 3 independent experts for review [4]. Each expert also received a set of reference materials created by the developers of Nova [10,11,13,28] and a handout adapted from previous publications about the Nova classification system to use during the grading process (Supplemental Figure 1) [[8], [13],^28^,^31^].

Individual food products (identified by their generic, non-branded names) and their ingredients were added to a Qualtrics survey as “questions” with Nova classifications of 1, 2, 3, and 4 as potential answers. Three experts (“graders”) familiar with the Nova classification system completed the survey. Scores for each food had to be agreed upon by 2 out of 3 external “graders” [4], a practice used in a previous publication with similar methods [18]. As an additional data quality measure to ensure graders thoroughly read each survey question when reviewing Nova classifications, 11 additional foods and beverages were added to the survey.

For recipes, which were the basis of many meals on the LPW, individual ingredients that made up each menu were included in the surveys provided to graders. Final Nova classifications for recipes were determined by the highest category score received by any individual ingredient in that recipe. Because the current publications on the Nova classification system do not provide clear guidance on how to determine categorization for homemade foods [4], we followed the same principles applied to prepared or packaged foods. That is, if there is even a single ingredient that functions as a cosmetic additive or that receives a score of 4 on the Nova classification system, the food is considered “ultra-processed.”

Food items with consensus scores from 2 of 3 Nova classification graders that placed them in Nova categories 1 or 2 were used as the basis of the LPW.

### Nutrient content of MPW and LPW

The macro- and micronutrient content of the LPW and MPW were determined using the USDA National Nutrient Database for Standard Reference, Legacy [32] through a customized in-house nutrient analysis program at the GFHNRC. Each menu item was individually matched with its equivalent in the proprietary analysis program after evaluation of ingredients, energy, macronutrient, and micronutrient content by trained research staff. Certain items that did not have an equivalent in this database, including dairy-free gravy, cassava-based puffed snacks, wedding cookies, homemade fruit punch, and almond flour pancake mix, were added separately using nutrition information specific to the exact products identified for the LPW.

### Food cost determination

Grocery retailer websites were used to calculate the average daily cost of both the LPW and the MPW. For foods and beverages that must be purchased in a larger container, even if only a portion of the ingredient would be used in a recipe (for example, yeast, flour, salt, etc.), the cost of the most frequently purchased container was used. For both the LPW and MPW, there is both a “per person” cost that reflects the cost of preparing each recipe for the menu (for example, 6 fl oz of juice, even if a 32 fl oz container is purchased) and the “true cost” that reflects the cost of buying the entire container. For both the MPW and LPW cost determinations, the “per person” calculation reflects only the cost of the ingredients used to prepare the menu for a single person. The LPW, for example, includes the cost of 241 g of lemons, which equates to roughly 2.23 lemons, even though it is not actually possible to purchase a fraction of a lemon.

All cost information for the menus was gathered between August and November 2023 from grocery retailers in the Northern Great Plains region of the United States. No sales or discounted prices were included. Wherever possible, the same brand and product were used on both menus (for instance, the same brand of eggs and salt were used in both menus), so cost differences reflect changes in costs due to selection of more- or less-processed items compared with comparing different brand prices for the same product.

Cost calculations per person also allowed us to calculate the amount of food leftover by subtracting the amount (g) used in the menu from the total container size (g). Because these items could be repurposed in another meal, this difference is labeled as “leftover food” instead of “food waste.”

### Shelf stability

To evaluate shelf stability of the MPW and the LPW, first, each food was designated as packaged or unpackaged food. Produce, recipes, and unprocessed meat/protein products (ground beef, eggs, chicken) were considered unpackaged foods.

The most closely related food category for each food or beverage item was identified on the FoodKeeper App (foodsafety.gov, developed by the United States Department of Health and Human Services) [33], and the time to expiration of each food or beverage item was recorded in alignment with how the product was purchased. That is, time to expiration if a product was shelf-stable, frozen, or refrigerated, assuming it stayed in its original format, was recorded. If no corresponding food or beverage item was listed on the FoodKeeper App, other resources were used, including the Greater Pittsburgh Community Food Bank Shelf Life manual [34] and publicly available refrigerator and freezer storage charts [[35], [36], [37]] to determine the maximum number of days of the product’s shelf-life (days to expiration).

### Statistical analysis

Tie-corrected Kendall’s coefficient of concordance (*W*) was used to quantify the inter-rater reliability of Nova classifications between graders [38], using the same process conducted in a previous study [18]. This statistical analysis was performed using R software [39] with the irr package [40]. To compare shelf stability, the Kaplan-Meier survival analysis method was used to obtain median time to expiration of the overall menus for LPW and MPW. The Wilcoxon homogeneity testing of survival curves was used to compare maximum shelf stability between the 2 diets. These analyses were performed using SAS 9.4 software. Copyright © 2023 SAS Institute Inc. SAS and all other SAS Institute Inc. product or service names are registered trademarks or trademarks of SAS Institute Inc. [41]. A significance level of α = 0.05 was set a priori.

## Results

The final LPW can be found in Supplemental Table 2.

### Nova classifications

Graders aligned on 11 out of 11 quality assurance items. A table listing a side-by-side comparison of the MPW and LPW can be found on Table 1. Agreement between graders for Nova classifications for items on LPW and MPW was high (*W* = 0.78, *P <* 0.001 for LPW; *W* = 0.92; *P <* 0.001 for MPW). The LPW menu includes ∼48% of kcal from Nova categories 1 and 2, whereas the MPW includes ∼21% of kcal from Nova categories 1 and 2. The LPW included 20% energy (kcal) from Nova category 4 foods (ultra-processed), whereas the MPW included 67% energy from UPFs. The amounts of energy from Nova categories can be found in Table 2. For some foods, all 3 graders provided different categorizations, so these items are considered as “unclassifiable energy” (Table 2). Details on the categorization of all foods in the LPW and MPW are provided in Supplemental Tables 3 and 4.

**TABLE 1:** Day 2 meals from more-processed Western, less-processed Western, and adjusted less-processed Western menus.

	MPW	LPW	aLPW
Breakfast	Grape juice cocktail Pancakes Butter Syrup Bacon	Grape juice Pancake mix Salted butter Pure maple syrup Uncured bacon	Grape juice Pancakes^1^ Salted butter Pure maple syrup Bacon^1^
Lunch	Lemonade Chicken burger White hamburger bun Miracle whip Breaded chicken patty Romaine lettuce Chips Cashews Dark chocolate	Lemonade Chicken sandwich Chicken strips Homemade hamburger buns Mayonnaise Iceberg lettuce White corn tortilla chips Salted cashews Milk chocolate	Lemonade Chicken sandwich Breaded chicken patty^1^ Homemade hamburger buns Miracle whip^1^ Iceberg lettuce White corn tortilla chips Salted cashews Dark chocolate^1^
Dinner	Chocolate 2% milk Hamburger hotdish Elbow macaroni Ground beef Canned diced tomatoes with green pepper, celery, and onion Condensed tomato soup Corn White dinner roll Butter	Whole milk Hamburger hotdish Elbow macaroni Ground beef Tomatoes Cream of tomato soup Corn Homemade dinner rolls Salted butter Homemade brownie	Whole milk Hamburger hotdish Elbow macaroni Ground beef Tomatoes Cream of tomato soup Corn Homemade dinner rolls Salted butter Homemade brownie

Abbreviations: aLPW, adjusted less-processed Western; LPW, less-processed Western; MPW, more-processed Western.

1 Denotes items changed from original LPW.

**TABLE 2:** Percentage of calories from NOVA categories (more- and less-processed Western menus).

	More-processed Western	Less-processed Western	Adjusted less-processed Western
Total 5-d energy average	2021 kcal/d	2201 kcal/d	2173 kcal/d
Group 1 and 2 foods (minimally processed, unprocessed, and processed culinary ingredients)	21.1% kcal	47.9% kcal	50.8% kcal
Group 3 foods (processed foods)	10.6% kcal	19.4% kcal	17.5% kcal
Group 4 foods (ultra-processed foods)	67.3% kcal	20.3% kcal	20.4% kcal
Energy from negligible ingredients	0.10% kcal^1^	0.3% kcal^3^	0.3% kcal^3^
Unclassifiable energy	0.80% kcal^2^	9.6% kcal^4^	9.7% kcal^4^

1 Poultry seasoning, onion powder, garlic powder.

2 Sour cream.

3 Taco seasoning mix, ground cinnamon, vanilla extract, Parmesan cheese, dried parsley, poultry seasoning, onion powder, garlic powder.

4 Prepared yellow mustard, cooked enriched macaroni, bottled grape juice, maple syrup, sour cream, egg noodles.

### Adjustments

Graders categorized more items on the LPW as category 4 foods (UPFs) than anticipated (Supplemental Table 5). Because the primary difference between the LPW and MPW was intended to be the relative percentages of energy coming from UPFs, we also generated an “adjusted” version of the LPW using the same category 4 foods from the MPW. This shift mitigates potential differences in cost, nutrient density, or shelf stability that may arise from having different UPFs in the LPW. Supplemental Table 6 lists the food items from the MPW that replaced items on the LPW. All subsequently discussed results will be listed both with the original LPW and with this “adjusted” LPW (aLPW), which is listed in its entirety in Supplemental Table 7.

Like the original LPW, the aLPW included 20% energy from category 4 foods and ∼51% kcal from Nova categories 1 and 2 (Table 2).

### Nutrient density

Table 3 lists the macro- and micronutrient content of the LPW, MPW, and aLPW menus as well as their nutrient density alongside the Dietary Reference Intakes for females aged 19–30 y and males aged 51 y and older, because these 2 populations are the 2 cited in the 2020 DGA as most likely to need a 2000-kcal/d eating pattern. The LPW and aLPW provide more energy, protein, and fat (including saturated fat) than the MPW but have 45% energy from carbohydrates compared with 50% on the MPW. The MPW has less calcium, more iron, less potassium, and less vitamin D than the LPW and aLPW.

**TABLE 3:** Macro and micronutrient content of less- and more-processed Western menus.

Nutrient	More-processed Western diet Average (min, max)	Low-processed Western diet Average (min, max)	Adjusted low-processed Western diet Average (min, max)	Dietary reference intakes, females 19–30 y	Dietary reference intakes, males 51+ y
Energy (kcals)	2034 (1980, 2074)	2178 (1746, 2410)	2142 (1755, 2343)	2000	2000
Protein (g)	74 (67, 82)	82 (71, 94)	82 (71, 95)	112	56
Carbohydrate (g)	252 (234, 281)	243 (160, 293)	242 (166, 288)	130	130
Fat (g)	83 (70, 94)	100 (87, 116)	97 (86, 116)	n/a	n/a
Protein (% kcals)	15 (13, 16)	14 (12, 20)	15 (13, 19)	10–35	10–35
Carbohydrate (% kcals)	50 (46, 55)	45 (37, 50)	45 (38, 51)	45–65	45–65
Fat (% kcals)	37 (32, 41)	41 (38, 45)	41 (37, 44)	20–35	20–35
SFA (% kcals)	13 (10, 17)	15 (13, 17)	14 (13, 18)	<10% total kcals	<10% total kcals
MUFA (% kcals)	12 (11, 13)	14 (12, 15)	14 (13, 16)	n/a	n/a
PUFA (% kcals)	9 (5, 12)	9 (6, 11)	9 (6, 12)	n/a	n/a
Dietary fiber (g)	14 (12, 17)	15 (11, 22)	14 (11, 20)	28	28
Calcium (mg)	603 (526, 682)	704 (493, 1053)	696 (503, 1032)	1000	1000; 1200 (for males over 71)
Copper (mg)	1.2 (1.0, 1.4)	1.3 (1.1, 1.5)	1.4 (1.0, 1.7)	0.9	0.9
Iron (mg)	18 (15, 24)	13 (12, 15)	16 (12, 21)	18	8
Magnesium (mg)	251 (210, 298)	275 (245, 289)	288 (245, 329)	310	420
Manganese (mg)	2.5 (1.7, 3.2)	3.4 (2.4, 4.6)	3.3 (2.2, 4.7)	1.8	2.3
Phosphorus (mg)	1200 (1033, 1493)	1400 (1108, 1872)	1373 (1134, 1763)	700	700
Zinc (mg)	11 (10, 13)	12 (12, 14)	12 (11, 14)	8	11
Potassium (mg)	2210 (1893, 2481)	2383 (2062, 2602)	2355 (2048, 2549)	2600	3400
Sodium (mg)	2689 (2459, 3013)	2777 (2017, 3386)	2826 (2154, 3486)	2300	2300
Vitamin C (mg)	115 (61, 192)	26 (15, 40)	26 (16, 41)	75	90
Thiamin (mg)	1.7 (1.2, 1.9)	1.7 (1.0, 2.0)	1.6 (1.0, 2.0)	1.1	1.2
Riboflavin (mg)	1.7 (1.3, 2.1)	2.3 (1.5, 2.8)	2.3 (1.5, 2.8)	1.1	1.3
Niacin (mg)	23 (17, 29)	22 (18, 29)	22 (17, 29)	14	16
Vitamin B_6_ (mg)	1.5 (1.4, 1.7)	1.6 (1.2, 2.0)	1.6 (1.2, 2.3)	1.3	1.7
Folate, DFE (mg)	444 (309, 592)	482 (406, 604)	489 (392, 570)	400	400
Vitamin B_12_ (mg)	4.0 (2.9, 5.1)	4.3 (3.3, 5.8)	4.7 (3.3, 7.4)	2.4	2.4
Vitamin A (mg RAE)	608 (392, 896)	724 (402, 1159)	722 (400, 1190)	700	900
Vitamin D (mg)	3.2 (1.6, 5.2)	4.6 (0.5, 9.1)	5.1 (0.6, 10)	15	15; 20 (for older than 71)
Vitamin E (mg AT)	8.0 (3.3, 12.8)	11.7 (5.1, 21.7)	8.4 (4.7, 13.7)	15	15
Carotene, alpha (mg)	398.9 (7.004, 1498)	382.7 (10.6, 1391)	380.1 (10.6, 1386)	n/a	n/a
Carotene, beta (mg)	1607 (543, 4204)	2141 (665, 4128)	2139 (655, 4125)	n/a	n/a
Cryptoxanthin (mg)	54 (12, 130)	33 (9, 57)	33 (9, 65)	n/a	n/a
Lycopene (mg)	5358 (0, 17624)	1825 (0.1, 8096)	1825 (0.1, 8096)	n/a	n/a
Lutein + Zeaxanthin (mg)	1268 (1029, 1953)	2627 (739, 8559)	2570 (623, 8469)	n/a	n/a

Abbreviations: AT, α-tocopherol; DFE, dietary folate equivalent; RAE, retinol activity equivalent.

### Diet quality (HEI-2015)

**FIGURE 1:**
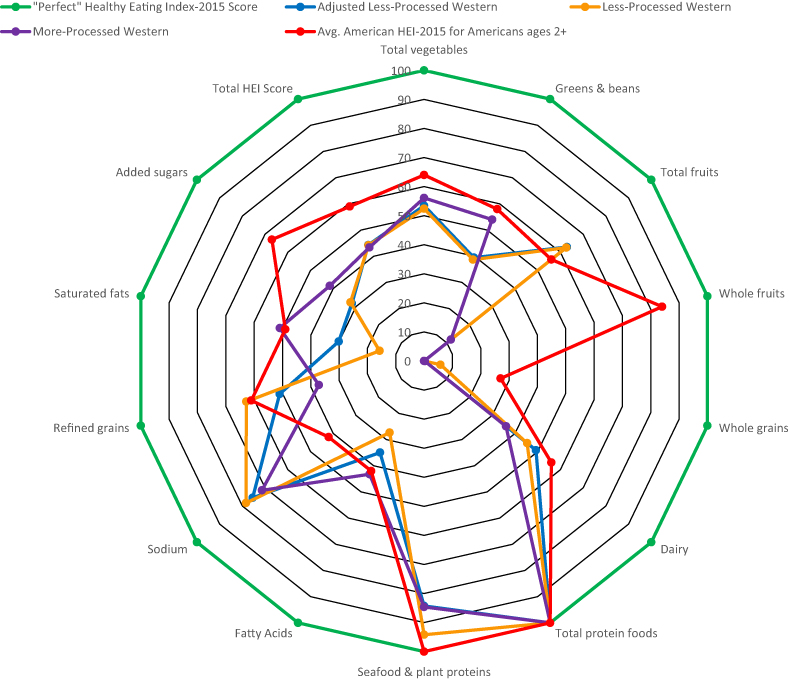
Radar plot depicting Healthy Eating-2015 (HEI-2015) scores for a more-processed Western menu, less-processed Western menu, average American eating pattern, and a perfect HEI-2015 score.

The HEI-2015 scores for the LPW and the aLPW were 44 out of 100, whereas the HEI-2015 score for the MPW is 43 out of 100. Figure 1 shows the HEI-2015 scores for the LPW, MPW, and aLPW and scores for each adequacy component. Supplemental Figure 2 compares the LPW and aLPW scores.

### Menu cost and leftover food calculations

The average cost of the MPW, created without attention to the degree of processing of the foods included in it, netted to $56.47/d. The average cost of the LPW was $79.46/d. After adjustments for UPF ingredients, the cost of the aLPW was $68.06/d. These amounts reflect the true cost of the diet, based on buying full containers of all ingredients. All cost information can be found in Supplemental Tables 8–10.

The “per person” cost, which reflects only the amount of the food used in the diet is $9.85/d for the MPW, $15.89 for the LPW, and $13.61/d for the aLPW. These amounts do not reflect the total cost, because they do not account for items that cannot readily be purchased in only the amounts needed for a specific recipe (such as flour, sugar, and other sundries).

Leftover food, which is the sum of food purchased but not used in the 5-d menu, totaled between 31 and 38 kg on the 3 menus (MPW, LPW, aLPW). The amount of leftover food includes all inedible and edible portions of food unused in the menu or recipes.

### Shelf stability

The LPW and aLPW had 39% kcals from packaged foods, whereas 63% kcals of the MPW came from packaged foods. In terms of the number of food items, 53% of individual food items on the LPW and aLPW were “packaged,” whereas 74% of individual food items on the MPW were packaged foods.

Relative percentages of the number of shelf-stable, frozen, and refrigerated food items were similar between the 2 diets (LPW and aLPW had 41% shelf-stable and 5% frozen foods; MPW had 50% shelf-stable and 7% frozen foods) (Table 4). Using the Kaplan-Meier survival analysis method, median time to expiration of the LPW menu items was 35 d compared with 120 d for the MPW menu items. The Wilcoxon homogeneity testing of survival curves for maximum shelf stability yielded a statistically significant difference (*P* = 0.0059) between the 2 diets. Details on shelf stability data of different foods in the MPW and LPW menus can be found in Supplemental Tables 11 and 12.

**TABLE 4:** Packaged, unpackaged, frozen, shelf-stable, and refrigerated foods on the more-processed Western menu, less-processed Western menu, and adjusted less-processed Western menu.

	LPW	MPW	aLPW
% kcals from packaged foods	39	63	39
% kcals from unpackaged foods	61	37	61
% packaged foods	53	74	53
% unpackaged foods	47	26	47
% shelf-stable foods	41	50	41
% frozen foods	5	7	5
% refrigerated foods	54	44	54

Abbreviations: aLPW, adjusted less-processed Western; LPW, less-processed Western; MPW, more-processed Western.

## Discussion

The results of this study indicate that menus with low diet quality measures can also be comprised primarily of less-processed foods, according to the Nova classification system. The more-processed menu in this study had a similar diet quality score, nutrient density, and amount of leftover food but a longer shelf stability and a lower cost compared with the LPW and aLPW. The 2 primary conclusions we draw from this study are that processing level, as defined by the Nova classification system, does not serve as a proxy indicator of diet quality and that, although they can provide equivalent nutrition to less-processed options, more-processed foods may have a longer shelf life and a lower cost.

The LPW and aLPW indicate that menus that include fewer processed foods, according to the Nova classification system, can still be of low quality, high in nutrients to limit (saturated fat and sodium), and low in nutrients to encourage (fiber, calcium, potassium). Encouraging or forcing consumers to decrease intake of processed foods is not a guarantee that consumers will then select more nutritious options.

In addition, processed foods including UPFs can provide accessible nutrient-dense options. Although shelf stability and safe storage is a topic of concern in discussions of food safety and the environmental impact of foods (in terms of food waste and energy needs to cook and store foods) [42], shelf stability and safe storage are infrequently evaluated as contributors to food access and food cost. To our knowledge, this is the first study that has assessed relative shelf stabilities of entire menus. Shelf-life of foods is especially important in the context of food access in the United States. A USDA Economic Research Service bulletin from 2015 found that the average United States household lives over 2 miles from the nearest supermarket [43]. Longer product shelf life may also be an important consideration for larger households, which are more likely to participate in fewer large shopping trips [44]. Consumer demand has spurred innovation for foods with longer shelf lives. A book published over 20 y ago detailed the challenge the food industry faced with developing minimally processed foods that simultaneously met consumer demands for longer shelf life [45]. This challenge persists today.

Some less-processed foods that provide similar nutrition to more-processed alternatives are more expensive or less shelf stable or both. A few examples from the menus in this study that are more expensive when less processed include cranberry juice, sunflower seeds, canned beans, and cured pork products. Several of the recipes on the LPW (tater tots, lemonade, dinner rolls, hamburger buns, brownies) are also less shelf stable than their more-processed counterparts on the MPW, but both the more- and less-processed versions of these foods provide similar nutrition. There is not currently intervention research that details a detriment to consuming more-processed versions of nutrient-dense products like canned beans, fluid milk, tofu, and oatmeal. Because more-processed foods had a longer shelf life in this study, processed foods may lead to decreased food waste because leftover foods may remain safe for longer. More research is needed on shelf stability and food waste with dietary patterns including more- and less-processed foods.

The cost of the aLPW aligns with the cost of the Liberal Food Plan (LFP; compared with the Thrifty Food Plan and Moderate-Cost Food plan) from the November 2023 release of the cost of food at home at 3 levels (United States average) [46]. Because a 2000-kcal diet may be appropriate for females aged 19–30 y and males aged 51+ y, we looked at how the LFP allots weekly grocery budgets for those 2 groups. A single female aged 20–50 eating the LFP costs $93.60/wk ($13.37/d), and a male aged 51–70 y eating the LFP costs $97.90/wk ($13.99/d) [46]. Although the LFP is intended to provide a healthy dietary pattern and the LPW and MPW do not, the cost of the LFP for both groups is within a few cents of the daily cost of the aLPW ($13.61/d) but is lower than the daily cost of the LPW ($15.89/d) and higher than the cost of the MPW ($9.85/d). However, the diet costs in this study do not reflect the cost of purchasing entire containers of ingredients, even when such a purchase would be required. In this study, the menu with less-processed options led to a costlier diet but with a daily cost in line with the LFP.

Finally, identifying foods by level of processing, especially using the Nova classification system, continues to pose challenges [3]. At the time of this writing, clear guidance is lacking from the Nova team on how to apply the system to recipes intended to be prepared at home. A system developed for use with the NHANES data that involves disaggregating foods to determine their Nova classifications does not function in the same way with recipes that consumers might use [47]. Also, the Nova classification system’s use of the term “homemade” for many products is challenging to implement, because there is no consistent usage of the term homemade by either researchers or consumers [48]. As noted by Mills et al. [48], “the definition of ‘home cooking’ [in Nova] excluded many convenience foods and ready-made products, though appeared to include widely used time-saving ingredients, such as dried pasta and tinned tomatoes.” Yet, both dried pasta and tinned tomatoes are not only ready-made products but can also be ultra-processed products, depending on their ingredient makeup. Therefore, it is unclear whether a pasta dish made with these products would be considered homemade (category 3) or ultra-processed (category 4). Despite these challenges, this study found high levels of agreement among graders for Nova classifications for both the LPW and MPW [18]. More work is needed to address these challenges with Nova classification system implementation among the scientific community, and research is also needed to understand how consumers understand the term “ultra-processed” and might apply this scale.

### Limitations

Like other studies that use the Nova classification system to categorize foods based on level of processing, using Nova requires creation of a specific menu compared with a dietary pattern. Careful implementation of Nova requires using specific brands for each food item, because the ingredient list can vary between brands even with the same product (such as canned pinto beans), which can then place the same food in different Nova categories. Using a menu with specific branded food products, however, limits the applicability of the findings on cost, shelf stability, nutrient density, and diet quality from this study. Other iterations of menus could be more or less cost-effective, nutrient dense, or shelf stable. The Nova classification system limits the scope of this study to a proof of concept and methodology development project. Future studies may use the methods used in this study to further investigate the impact of processing level on shelf stability at a diet level.

The cost data used in this study have limited applicability as well for several reasons. The costs in this study primarily reflect purchasing options at a local grocery chain during a period of inflation. For a few food items (100% cranberry juice, cassava puffs, Mexican wedding cookies, taco seasoning), price was derived from a national grocery chain during the same time frame (Fall 2023). Although nationally representative cost data were available from ERS’s Purchase to Plate tool [49], this dataset provides updates through 2018 and does not include branded food items. Using actual grocery store prices limits the applicability of the findings of this study; however, the results represent a realistic estimate of the costs of purchasing ingredients for the LPW or MPW in Fall 2023. The cost estimates are based on the most purchased size of each container rather than the smallest amount needed for use in the study menus. For instance, the cost in the menus reflects the cost of a 32 fl oz container of juice, even if only 6 fl oz of juice was used in the menus. A smaller container of juice would decrease the cost of these diets even if the unit price would likely increase from purchase of a smaller container size.

In conclusion selecting less-processed foods according to the Nova classification system does not guarantee a high-quality diet. In this study, we used the Nova classification system to design a Western diet with less-processed foods to match the meals of a Western diet with more-processed foods. Using less-processed foods did not improve the nutritional value of the diet and resulted in projected greater costs and a shorter shelf life than the comparable menu with more-processed foods. Applying the Nova classification system, the most applied framework for determining whether a food is “ultra-processed” in dietary guidance, would not necessarily simplify the process of selecting more nutritious diets for consumers and may also increase food cost as well as food waste.

## Author contributions

The authors’ responsibilities were as follows— JMH: designed the study; MEC, AJS, AB, ASL: conducted the research; JMH, MEC: analyzed the data and wrote the first manuscript draft; all authors: read and approved the final manuscript.

## Data availability

Data described in the manuscript will be made publicly and freely available without restriction at Open Science Framework (OSF): https://osf.io/fykwt/?view_only=cb6d368a3ada4b4280a6fbbe6c99621f.

## Funding

USDA Agricultural Research Service project grant #3062-51000-057-00D.

## Conflict of interest

The authors report no conflicts of interest.
