# Supplementary material for: Using Less Processed Food to Mimic a Standard American Diet Does Not Improve Nutrient Value and May Result in a Shorter Shelf Life at a Higher Financial Cost
Source: Curr Dev Nutr. 2024 Oct 2;8(11):104471. doi: 10.1016/j.cdnut.2024.104471 (PMC11539364; doi:10.1016/j.cdnut.2024.104471)
Supplement: Supplementary file 1 — Multimedia component 1 [file mmc1.docx]

# **Title:** Using less processed food to mimic a standard American diet (SAD) does not improve nutrient value and may result in a shorter shelf life at a higher financial cost

# **First Author:** Hess, Julie M

# Online Supplementary Material – Supplemental Tables

# Supplemental Table 1. 2000 kcal More-Processed Western 5-Day Menu

| **DAY 1 BREAKFAST** | **DAY 2 BREAKFAST** | **DAY 3 BREAKFAST** | **DAY 4 BREAKFAST** | **DAY 5 BREAKFAST** |
| --- | --- | --- | --- | --- |
| Cran-Rasp Juice Cocktail  Ham & Cheese Egg Bake  Crispy Crowns  White Bread  Butter  Strawberry Jelly | Grape Juice Cocktail  Pancakes  Butter  Syrup  Bacon | Peach Punch  Scrambled Eggs  Shredded Cheddar  White Bread  Butter  Strawberry Jelly | Cran-Rasp Juice Cocktail  Baked French Toast  Custard Mix  Texas Bread  Butter  Syrup  Bacon | Grape Juice Cocktail  1% Milk  Frosted Corn Flakes  White Bread  Sunflower Butter  Honey |
| **DAY 1 LUNCH** | **DAY 2 LUNCH** | **DAY 3 LUNCH** | **DAY 4 LUNCH** | **DAY 5 LUNCH** |
| Fruit Punch  Taco Salad  Taco Meat  Iceberg Lettuce  Grape Tomatoes  Shredded Cheddar  Sour Cream  Salsa  Tortilla Chips  Chocolate Cookies | Lemonade  Chicken Burger  White Hamburger Bun  Miracle Whip  Breaded Chicken Patty  Romaine Lettuce  Chips  Cashews  Dark Chocolate | Chocolate 2% Milk  Chili  Chili Beans  Ground Beef  Celery  Salsa  Sunflower Kernels  Cornbread  Honey  Cashew Shortbread | Fruit Punch  Pizza Hotdish  Spiral Macaroni  Ground Beef  Pepperoni  Pizza Sauce  Shred Mozzarella  Garlic Breadstick  Cashews  Chocolate Cookies | Lemonade  Ham Sandwich  White Hamburger Bun  Mustard  Sliced Ham  Iceberg Lettuce  Macaroni Salad  Elbow Macaroni  Carrots  Celery  Peas  Green Onions  Miracle Whip  Mustard  Chips  Dark Chocolate |
| **DAY 1 DINNER** | **DAY 2 DINNER** | **DAY 3 DINNER** | **DAY 4 DINNER** | **DAY 5 DINNER** |
| Peach Punch  Chicken & Stuffing  Stuffing  Butter  Chicken  Chicken Gravy  Green Beans  Sunflower Kernels  White Dinner Roll  Butter  Brownie | Chocolate 2% Milk  Hamburger Hotdish  Elbow Macaroni  Ground Beef  Canned diced tomatoes with green pepper, celery, and onion  Condensed Tomato Soup  Corn  White Dinner Roll  Butter | Fruit Punch  Pork & Noodles  Egg Noodles  Pork Loin  Sour Cream  Beefy Mushroom Soup  Peas  Steamed Petite Carrots  Brownie | Chicken & Potatoes  Red Potatoes  Chicken  Chicken Gravy  Chicken Seasoning  Peas  White Dinner Roll  Butter  Crispy Rice Bar | Chocolate 2% Milk  Tator Tot Hotdish  Ground Beef  Corn  Condensed Mushroom Soup  Onion Powder  Garlic Powder  Crispy Crowns  White Dinner Roll  Butter  Cashew Shortbread |

# Supplemental Table 2. 2000 kcal Less-Processed Western 5-Day Menu

| **DAY 1 BREAKFAST** | **DAY 2 BREAKFAST** | **DAY 3 BREAKFAST** | **DAY 4 BREAKFAST** | | **DAY 5 BREAKFAST** |
| --- | --- | --- | --- | --- | --- |
| Cranberry Juice  Prosciutto Egg Cups  Homemade White Bread  Salted Butter  Berry jam without pectin | Grape Juice  Pancake Mix  Salted Butter  Pure Maple Syrup Uncured Bacon | Vanilla Steamer  Scrambled Eggs  Homemade White Bread  Salted Butter  Berry jam without pectin | Apple Juice  Ricotta Stuffed French Toast  Salted Butter  Maple Syrup  Uncured Bacon | | Grape Juice  Almond Steamer  Simple Syrup  Brown Sugar Maple Instant Oatmeal  Homemade White Bread  Sunflower Butter  Honey |
| **DAY 1 LUNCH** | **DAY 2 LUNCH** | **DAY 3 LUNCH** | **DAY 4 LUNCH** | | **DAY 5 LUNCH** |
| Fruit Punch  Taco Salad  Taco Meat  Iceberg Lettuce  Grape Tomatoes  Shredded pepperjack  Sour Cream  Mild Salsa  White Corn Tortilla Chips | Lemonade  Chicken Sandwich  Chicken Strips  Homemade Hamburger Buns  Mayonnaise  Iceberg Lettuce  White Corn Tortilla Chips  Salted Cashews  Milk Chocolate | Whole Milk  Chili  Slow Cooker Beans  Ground Beef  Celery  Mild Salsa  Sunflower Seeds  Cornbread Mix  Honey  Mexican Wedding Cookies | Fruit Punch  Pizza Hotdish  Spiral Macaroni  Ground Beef  Turkey pepperoni  Pizza Sauce  Shredded Mozzarella  Homemade Breadsticks  Salted Whole Cashews  Mexican Wedding Cookies | | Lemonade  Ham Sandwich  Homemade Hamburger Buns  Mustard  Ham  Iceberg Lettuce  Macaroni Salad  Elbow Macaroni  Carrots  Celery  Peas  Green Onions  Mayonnaise  Mustard  Casava Puffs  Dark Coconut Toffee Organic Chocolate |
| **DAY 1 DINNER** | **DAY 2 DINNER** | **DAY 3 DINNER** | **DAY 4 DINNER** | | **DAY 5 DINNER** |
| Lemonade  Chicken & Stuffing  Stuffing Recipe  Salted Butter  Sunflower Seeds  Chicken  Gravy  Green Beans  Homemade Dinner Rolls  Salted Butter  Homemade Brownie | Whole Milk  Hamburger Hotdish  Elbow Macaroni  Ground Beef  Tomatoes  Cream of Tomato Soup  Corn  Homemade Dinner Rolls  Salted Butter  Homemade Brownie | Fruit Punch  Pork & Noodles  Egg Noodles  Pork Tenderloin  Sour Cream  Organic Cream of Mushroom Soup  Peas  Steamed Carrots  Homemade Brownie | Chicken & Potatoes  Red Potatoes  Chicken  Gravy  Chicken Seasoning  Peas  Homemade Dinner Rolls  Salted Butter  Homemade Crispy Rice Treats | | Whole Milk  Tator Tot Hotdish  Ground Beef  Corn  Cream of Mushroom Soup  Onion Powder  Garlic Powder  Homemade tater tots  Homemade dinner rolls  Salted Butter  Mexican Wedding Cookies |
|  | | | |  |  |

# Supplemental Table 3. Foods in Less-Processed Western Menu by NOVA Category

## Category 1

| **Day/Meal** | **Food Description** | **Ingredients** | **Manufacturer/Product Name** |
| --- | --- | --- | --- |
| Day 1/Breakfast (B) | 100% Cranberry Juice | Cranberry juice (water, cranberry juice concentrate) | Ocean Spray |
| Day 1/B | Spinach, raw | Spinach | Dole |
| Day 1/B | Egg, whole, cooked, poached | Eggs | Essential Everyday |
| Day 1/L | Beef, ground, 93% lean meat/ 7% | Ground beef | Hugo’s |
| Day 1/L | Fruit Punch* | Water, grape juice, plain orange juice/orange carrot juice, apple juice/apple cider | n/a |
| Day 1/L | Cherry Tomatoes | Tomatoes | Hugo’s |
| Day 1/S | Boneless, skinless chicken breast | Chicken breast meat | Just Bare |
| Day 1/S | Green beans | Green beans, frozen | Birds Eye |
| Day 2/L | Shredded lettuce | Shredded lettuce | Dole |
| Day 2/S | Whole milk | Organic Grade A milk, Vitamin D3 | Organic Valley |
| Day 2/S | Tomato | Tomato | Hugo’s |
| Day 2/S | Frozen Corn | Corn | Birds Eye |
| Day 3/L | Dried Pinto Beans | Pinto beans | Essential Everyday |
| Day 3/L | Celery | Celery | n/a |
| Day 3/S | Pork loin | Pork | Hormel |
| Day 3/S | Frozen green peas | Green peas | Essential Everyday |
| Day 3/S | Petite carrots | Carrot | Bolthouse Farms |
| Day 4/B | Apple Juice | Water, apple juice concentrate, ascorbic acid | Mott’s |
| Day 4/S | Red potatoes | Red potatoes | Hugo’s |
| Day 4/S | Rice Cereal | Puffed brown rice | Arrowhead Mills |
| Day 5/L | Green Onion | Green onion | n/a |

## Category 2

| **Day/Meal** | **Food Description** | **Ingredients** | **Manufacturer/Product Name** |
| --- | --- | --- | --- |
| Day 1/B | Butter, salted | Pasteurized Cream, Salt | Kerry Gold |
| Day 1/B | Berry jam without pectin* | fresh berries, granulated sugar, lemon juice, salt | n/a |
| Day 1/B | Bread, white, prepared from recipe* | Instant yeast, flour, sugar, water, milk, butter | n/a |
| Day 1/S | Lemonade* | Sugar, water, lemon zest, lemon juice | n/a |
| Day 1/S | Dinner rolls* | Flour, sugar, yeast, butter, egg, salt | n/a |
| Day 1/S | Homemade Brownies* | Flour, eggs, white sugar, brown sugar, butter, cocoa powder, vanilla, flour, salt | n/a |
| Day 3/L | Honey | Honey | Essential Everyday |
| Day 4/L | Homemade Breadsticks* | Water, yeast, granulated sugar, salt, olive oil, flour, butter, salt, garlic power, parmesan cheese, Italian seasoning | n/a |
| Day 4/S | Simple Sugar* | White sugar, water | n/a |
| Day 5/S | Homemade tater tots* | Russet potatoes, butter, thyme/rosemary, flour, salt, pepper | n/a |
| Day 2/L | Hamburger buns* | Flour, sugar, yeast, butter, egg, salt | n/a |

*indicates recipe

Underlined ingredient(s) received highest NOVA rating

## Category 3

| **Day/Meal** | **Food Description** | **Ingredients** | **Manufacturer/Product Name** |
| --- | --- | --- | --- |
| Day 1/B | Ham, prosciutto | Pork, sea salt | Daniele Del Duca Prosciutto |
| Day 1/L | Pepper jack cheese | Pepper jack cheese | Crystal Farms |
| Day 1/L | Salsa | Tomatoes, onions, banana peppers, red bell peppers, green bell peppers, vinegars, water, salt, jalapeno peppers, garlic, spices | Curt’s Salsa |
| Day 1/L | White Corn Tortilla Chips | Corn, vegetable oil (corn, canola, and/or sunflower oil), salt | Santitas |
| Day 1/S | Sunflower seeds | Sunflower seeds, salt | Giants |
| Day 2/L | Cashews | Cashews, sea salt, peanut oil | Planters |
| Day 2/S | Cream of Tomato Soup | Organic Tomato Puree, Filtered Water, Organic Cream, Organic Cane Sugar, Organic Onions, Sea Salt, Organic Black Pepper. | Amy’s |
| Day 3/B | Scrambled Eggs* | Eggs, diced ham, cheddar cheese, heavy whipping cream, butter | n/a |
| Day 4/B | Ricotta Stuffed French Toast* | Easy homemade white bread (instant yeast, flour, sugar, water, milk, butter), ricotta cheese, eggs, half and half, vanilla, nutmeg, cinnamon, butter, confectioners’ sugar | n/a |
| Day 4/B | Ricotta cheese | Organic whole milk, organic skim milk, organic vinegar, salt | Organic Valley |
| Day 4/L | Rotini enriched macaroni | Semolina (wheat), niacin, Ferrous Sulfate (iron), thiamin mononitrate, riboflavin, folic acid | Essential Everyday |
| Day 4/L | Pizza Sauce | Italian Whole Peeled Tomatoes, Italian Cherry Tomatoes, Olive Oil, Fresh Carrots, Fresh Onions, Salt, Dried Oregano, Fresh Garlic, Fresh Basil. | Rao’s |
| Day 4/L | Mozzarella cheese | Low-moisture part-skim mozzarella cheese (cultured pasteurized reduced fat milk, salt, enzymes) | Essential Everyday |
| Day 4/S | Peanut butter, smooth | Peanuts, less than 1%: salt | Smucker’s |
| Day 4/S | Chocolate chips | Cane sugar, unsweetened chocolate, cocoa butter | Enjoy Life |
| Day 5/B | Sunflower butter | Roasted sunflower seeds, sugar, salt | SunButter |
| Day 5/L | Black Forest Ham | Organic pork (pork never administered antibiotics or animal by-product), water, sea salt, contains less than 2% of the following: organic cane sugar, cultured celery powder | Applegate |

## Category 4

| **Day/Meal** | **Food Description** | **Ingredients** | **Manufacturer/Product Name** |
| --- | --- | --- | --- |
| Day 1/S | Stuffing* | Homemade white bread (instant yeast, flour, sugar, water, milk, butter), yellow onion, celery, thyme leaves, ground sage, rosemary, salt, pepper, chicken/veggie broth | n/a |
| Day 1/S | No Dairy Gravy | Water, Organic Coconut Cream, Organic Turkey Bone Broth, Organic Tapioca Starch, Organic Coconut Aminos (Organic Coconut Blossom Nectar, Water, Himalayan Salt), Organic Chicken Fat, Organic Nutritional Yeast, Sea Salt, Organic Onion Powder, Poultry Spice Blend (Thyme, Sage, Marjoram, Rosemary, Black Pepper, Nutmeg), Organic Garlic Powder, Organic Turmeric. | Primal Kitchen |
| Day 2/B | Uncured bacon | Pork, water, sea salt, Less than 2%: cane sugar, celery powder | Applegate |
| Day 2/B | Pancakes | Organic Wheat Flour, Organic Cane Sugar, Organic Expeller-Pressed Sunflower Oil, Baking Powder (baking soda, sodium acid pyrophosphate, monocalcium phosphate), Sea Salt | Annie’s |
| Day 2/L | Chicken tenders, breaded, frozen | Organic White Meat Chicken (Chicken Never Administered Antibiotics or Fed Animal By-Products), Organic Wheat Flour, Water, Contains Less than 2% of the Following: Sea Salt, Organic Cane Sugar, Organic Paprika, Yeast, Organic Garlic Powder, Organic White Pepper, Breading Set in Organic Expeller Pressed Canola Oil. | Applegate |
| Day 2/L | Mayonnaise | Organic Chosen Blend Oil (Organic High Oleic Safflower Oil, Organic Avocado Oil, Organic Coconut Oil), Filtered Water, Organic Egg Yolks, Organic Whole Eggs, Organic Distilled White Vinegar, Organic Mustard (Distilled Organic Vinegar, Water, Organic Mustard Seed, Salt, Organic Spices), Salt, Organic Rosemary Extract. | Chosen Foods |
| Day 2/L | Coconut toffee chocolate bar | Organic Raw Cane Sugar (Fair Trade Certified by Fair Trade USA, Total 89.7% Fair Trade Ingredients), Organic Cacao Beans (Fair Trade Certified by Fair Trade USA, Total 89.7% Fair Trade Ingredients), Coconut Toffee (Organic Raw Cane Sugar (Fair Trade Certified by Fair Trade USA, Total 89.7% Fair Trade Ingredients), Organic Dessicated Coconut (Fair Trade Certified by Fair Trade USA, Total 89.7% Fair Trade Ingredients), Organic Butter, Sea Salt), Organic Cocoa Butter (Fair Trade Certified by Fair Trade USA, Total 89.7% Fair Trade Ingredients), Organic Whole Milk, Organic Butterfat. | Alter Eco |
| Day 3/L | Cornbread Mix | Whole Grain Cornmeal, Whole Wheat Flour, Sugar, Buttermilk Powder, Salt, Baking Powder (Sodium Acid Pyrophosphate, Sodium Bicarbonate, Cornstarch, Monocalcium Phosphate), Baking Soda. | Bob’s Red Mill |
| Day 3/L | Mexican Wedding Cookies | Grain Free Flour Blend (Almond Flour, Tapioca Flour, Arrowroot Flour, Cassava Flour, Chickpea Flour), Coconut Oil, Coconut Sugar, Powdered Sugar (Sugar, Tapioca Flour), Pecan Pieces, Avocado Oil, Apple Cider Vinegar, Sea Salt, Baking Soda, Cinnamon, Orange Peel Powder, Vanilla Extract, Jasmine Tea Extract | Siete |
| Day 3/L | Cream of Mushroom Soup | Filtered Water, Organic Mushrooms, Organic Onions, Organic Unbleached Wheat Flour, Organic High Oleic Safflower and/or Sunflower Oil, Organic Leeks, Organic Butter (Organic Cream, Salt), Organic cream, Organic Spices, Sea Salt, Organic Bay Leaves, Organic Black Pepper. | Amy’s |
| Day 4/L | Turkey Pepperoni | Turkey (Turkey Raised on Vegetarian Feed, on Family Farms, with at Least 33% More Space than Industry Standard and Environmental Enrichments to Promote Natural Behaviors and Well-Being), Sea Salt, Contains Less than 2% of the Following: Turbinado Sugar, Water, Spices, Cultured Celery Powder, Granulated Garlic, Cherry Powder, Lactic Acid Starter Culture (Not from Milk), Rosemary Extract, Oleoresin of Paprika, Lime Juice Concentrate. | Applegate |
| Day 5/B | Brown Sugar Maple Instant Oatmeal | Gluten Free Whole Grain Rolled Oats*, Brown Sugar*, Cane Sugar*, Maple Sugar*, Rolled Amaranth*, Rolled Quinoa*, Sorghum Flour*, Natural Maple Flavor, Sea Salt. *Organic. | Nature’s Path |
| Day 5/L | Salty snack puffs | Organic Cassava Flour, Organic Coconut Oil, Organic Tapioca Starch, Organic Coconut Flour, Organic Sweet Potato Flour, Himalayan Salt. | Lesser Evil |

# Supplemental Table 4. Foods in More-Processed Western Menu by NOVA Category

## Category 1

| Day/Meal | Food Description | Ingredients | Manufacturer/Product Name |
| --- | --- | --- | --- |
| Day 1/B | Egg, whole, raw, fresh | Large Egg | Essential Everyday |
| Day 1/B | 1% milk | \|  \| Low Fat Milk, Vitamin A Palmitate, and Vitamin D3 \| \| --- \| --- \| | Cass Clay |
| Day 1/L | Ground Beef Taco Meat | Ground beef,  Yellow Corn Flour, Salt, Maltodextrin, Paprika, Spices, Modified Corn Starch, Sugar, Citric Acid, Yeast Extract, Natural Flavors, Rosemary and Green Tea Extract, Silicon Dioxide. | Ortega (taco seasoning)  Cargill (beef) |
| Day 1/L | Lettuce | Iceberg lettuce | Dole |
| Day 1/L | Grape tomatoes | Grape tomatoes | Hugo’s Produce |
| Day 1/S | Chicken | Organic chicken breast meat. | Miller |
| Day 1/S | Frozen Green Beans | Green beans | Green Giant |
| Day 2/S | Corn | Corn | Birds Eye |
| Day 3/L | Celery | Celery | Dole |
| Day 3/S | Frozen Peas | Peas | Birds Eye |
| Day 3/S | Carrots | Carrots | Hugo’s Produce |
| Day 4/S | Red Potatoes | Red potato | Hugo’s Produce |
| Day 5/L | Green Onion | Green Onions | Hugo’s Produce |
| Day 5/S | Onion Powder | Onion | Essential Everyday |

## Category 2

| Day/Meal | Food Description | Ingredients | Manufacturer/Product Name |
| --- | --- | --- | --- |
| Day 1/B | Butter, salted | Pasteurized Cream, Salt | Kerry Gold |
| Day 1/L | Salsa, mild | Diced Tomatoes In Tomato Juice, Crushed Tomatoes (Water, Crushed Tomato Concentrate), Water, Jalapeno Peppers, Onions, Garlic, Distilled Vinegar, Dehydrated Onions, Salt, Cilantro, Natural Flavoring. | Pace |
| Day 3/L | Honey | Honey | Essential Everyday |
| Day 4/B | *Custard Mix | eggs, vanilla extract, brown sugar, cinnamon, butter, 1% milk, salt | N/A |
| Day 4/S | Poultry Seasoning | Garlic, Onion, Salt, Spices and Herbs (Including Black Pepper, Parsley, Red Pepper, Paprika), Orange Peel, and Green Bell Pepper. | McCormick |
| Day 5/S | Garlic Powder | Garlic | Essential Everyday |

*indicates recipe

Underlined ingredient(s) received highest NOVA rating

## Category 3

| Day/Meal | Food Description | Ingredients | Manufacturer/Product Name |
| --- | --- | --- | --- |
| Day 1/L | Tortilla Chips/Rounds | Corn, Vegetable Oil (Corn, Canola, and/or Sunflower Oil), and Salt. | Frito Lay (Tostito’s) |
| Day 2/L | Cashews | Cashews, Vegetable Oil (Peanut, Cottonseed, Soybean and/or Sunflower Seed), Sea Salt. | Essential Everyday |
| Day 2/S | Macaroni | Semolina (Wheat), Durum Flour (Wheat), Niacin, Iron (Ferrous Sulfate), Thiamin Mononitrate, Riboflavin, Folic Acid. | Creamette |
| Day 3/S | Egg Noodles | Durum flour (wheat), dried egg yolks or eggs, niacin, ferrous sulfate (iron), thiamin mononitrate, riboflavin, folic acid. | Creamette |
| Dat 3/S | Pork Loin | Pork: Contains Up to 12% Added Solution of Water, Potassium Lactate, Sodium Phosphates, Salt, Sodium Diacetate | Hormel |

## Category 4

| Day/Meal | Food Description | Ingredients | Manufacturer/Product Name |
| --- | --- | --- | --- |
| Day 1/Breakfast (B) | Cran-Raspberry Juice Cocktail | Filtered water, cranberry and raspberry juices from concentrate, natural flavors, citric acid, ascorbic acid (vitamin C), fruit and vegetable juice (color), and sucralose. | Langers |
| D1/B | Ham, sliced, packaged | Cured With Wheat Dextrose, Contains 2% Or Less Of Salt, Potassiuim Lactate, Sodium Lactate, Sodium Phosphates, Sodium Diacetate, Sodium Erythorbate, Sodium Nitrite May Contain Honey, Sugar | John Morrell |
| Day 1/B | Cheddar Cheese | Cheddar Cheese (Cultured Pasteurized Milk, Salt, Enzymes, Color Added), Anticake (Potato Starch, Powdered Cellulose), Natamycin (Natural Mold Inhibitor). | Essential Everyday |
| Day 1/B | Crispy Crowns | Potatoes, Vegetable Oil (Contains One of More of the Following: Soybean Oil, Canola Oil), Yellow Corn Flour, Salt, Granulated Onion, Spice Extractives, Dextrose and Disodium Dihydrogen Pyrophosphate to Promote Color Retention. | Essential Everyday |
| Day 1/B | Strawberry Jelly | Strawberry Juice, High Fructose Corn Syrup, Corn Syrup, Fruit Pectin, Citric Acid. | Smucker’s |
| Day 1/L | Chocolate Sandwich Cookie | Unbleached Enriched Flour (Wheat Flour, Niacin, Reduced Iron, Thiamine Mononitrate {Vitamin B1}, Riboflavin {Vitamin B2}, Folic Acid), Sugar, Palm and/or Canola Oil, Cocoa (Processed with Alkali), Invert Sugar, Leavening (Baking Soda and/or Calcium Phosphate), Soy Lecithin, Salt, Chocolate, Natural Flavor. | Nabisco |
| Day 1/L | Fruit Punch | Pure filtered water, cane sugar, pineapple, cherry, and cranberry juices, lemon juice (for tartness), cherry puree, natural flavors | Simply |
| Day 1/S | Sunflower Seeds | Sunflower Seed Kernels, Contains 2% or less of Gelatin, Sea Salt, Sugar, Cornstarch, Torula Yeast, Maltodextrin, Corn Syrup Solids, Paprika, Spices (Contains Celery Seed), Natural Smoke Flavoring, Onion Powder, Garlic Powder, Salt. May Contain: Peanuts, Tree Nuts. | Planters |
| Day 1/S | Gravy | Chicken Broth, Modified Food Starch, Chicken Fat, Contains Less than 2% of Bleached Wheat Flour, Salt, Yeast Extract, Canola Oil, Whey (from Milk), Natural Flavor, Onion Powder, Black Pepper, Spices, Dextrose, Fruit Juice (Color) Turmeric (Color), Oleoresin Paprika (Color). | Heinz |
| Day 1/S | Dinner Roll | Enriched Unbleached Flour (Wheat Flour, Malted Barley Flour, Niacin, Reduced Iron, Thiamine Mononitrate, Riboflavin, Folic Acid), Water, High Fructose Corn Syrup, Yeast, Contains Less than 2% of the Following: Soybean Oil, Salt, Wheat Gluten, Dough Conditioners (Sodium Stearoyl Lactylate, Monoglycerides, Ascorbic Acid, Calcium Sulfate, Enzymes), Monocalcium Phosphate, Calcium Propionate (Preservative). | Village Hearth |
| Day 1/S | Brownie Mix | Sugar, Enriched Bleached Wheat Flour (Flour, Niacin, Reduced Iron, Thiamine Mononitrate, Riboflavin, Folic Acid), Cocoa Powder Processed with Alkali, Vegetable Shortening (Palm Oil and/or Partially Hydrogenated Soybean Oil). Contains 2% or less of: Wheat Starch, Dextrose, Salt, Artificial Flavor, Carrageenan, Leavening (Sodium Bicarbonate). | Duncan Hines |
| Day 1/S | Stuffing Mix | enriched wheat flour (wheat flour, niacin, reduced iron, thiamin mononitrate [vitamin b1], riboflavin [vitamin b2], folic acid), high fructose corn syrup, onion*, contains less than 2% of salt, hydrolyzed soy protein, yeast, interesterified soybean oil, cooked chicken*, celery*, monosodium glutamate, parsley*, spice, maltodextrin, potassium chloride, sugar, chicken broth*, turmeric (color), silicon dioxide as an anticaking agent, disodium guanylate, disodium inosinate, natural flavor, with bha, bht and rosemary extract as preservatives.*dried | Kraft (Stove Top) |
| Day 1/S | Peach Punch | Pure filtered water, high fructose corn syrup, apple and peach juices from concentrate, less than 0.5% of: natural flavors, citric acid (provides tartness), vegetable and fruit juices (for color), pectin, sucralose, acesulfame potassium. | Minute Maid |
| Day 2/B | Grape Juice | Filtered Water, High Fructose Corn Syrup, Concord Grape Juice From Concentrate (Filtered Water, Concord Grape Juice Concentrate), Citric Acid (For Tartness), Grape Juice Concentrate (For Color), Ascorbic Acid (Vitamin C), Natural Flavor | Welch’s |
| Day 2/B | Pancakes | Enriched flour (wheat flour, malted barley flour, niacin, reduced iron, vitamin B1 [thiamin mononitrate], vitamin B2 [riboflavin], folic acid), water, high fructose corn syrup, vegetable oil (soybean and/or canola), buttermilk, contains 2% or less of leavening (baking soda, sodium aluminum phosphate, monocalcium phosphate), salt, soy lecithin, eggs. Vitamins and Minerals: Iron, vitamin A palmitate, vitamin B6 (pyridoxine hydrochloride), vitamin B12. | Kellogg’s |
| Day 2/B | Bacon | Cured with Water, Salt, Sugar, Sodium Erythorbate, Sodium Nitrite. | Hormel Black Label |
| Day 2/B | Syrup | Corn syrup, liquid sugar (natural sugar, water), water, salt, natural and artificial flavors (lactic acid), cellulose gum, preservatives (sorbic acid, sodium benzoate), sodium hexametaphosphate, caramel color, phosphoric acid. | Log Cabin |
| Day 2/L | Hamburger bun | Enriched Unbleached Flour (Wheat Flour, Malted Barley Flour, Niacin, Reduced Iron, Thiamine Mononitrate, Riboflavin, Folic Acid), Water, High Fructose Corn Syrup, Yeast, Wheat Gluten, Potato Flakes, Contains Less than 2% of the Following: Sugar, Soybean Oil, Salt, Calcium Propionate (Preservative), Dough Conditioners (Monoglycerides, Sodium Stearoyl Lactylate, Ascorbic Acid, Calcium Sulfate, Enzymes), Monocalcium Phosphate | Village Hearth |
| Day 2/L | Miracle whip | Water, soybean oil, high fructose corn syrup, vinegar, modified cornstarch, eggs, salt, natural flavor, mustard flour, potassium sorbates A preservative, paprika, spice, dried garlic. Contains: egg. | Kraft |
| Day 2/L | Chicken Patty | Chicken patties (chicken, water, salt, autolyzed yeast extract, sodium phosphates, natural flavorings), breader (bleached wheat flour, salt, dextrose, spice, soybean oil, yeast, extractives of paprika), batter (water, yellow corn flour, corn starch, salt, spices, guar gum, leavening [sodium acid pyrophosphate, sodium bicarbonate, monocalcium phosphate], garlic powder). Fried in vegetable oil. | Banquet |
| Day 2/L | Doritos | corn, canola, and/or sunflower oil), maltodextrin (made from corn), salt, cheddar cheese (milk, cheese cultures, salt, enzymes), whey, monosodium glutamate, buttermilk, romano cheese (part-skim cow's milk, cheese cultures, salt, enzymes), whey protein concentrate, onion powder, corn flour, natural and artificial flavor, dextrose, tomato powder, lactose, spices, artificial color (yellow 6, yellow 5, and red 40), lactic acid, citric acid, sugar, garlic powder, skim milk, red and green bell pepper powder, disodium inosinate, and disodium guanylate. | Frito Lay |
| Day 2/L | Lemonade | Pure filtered water, lemon juice from concentrate, high fructose corn syrup, less than 1% of: lemon pulp, natural flavors, pectin, sucralose, acesulfame potassium. | Minute Maid |
| Day 2/L | Dark Chocolate | Chocolate, sugar, cocoa butter, soy lecithin (emulsifier), bourbon vanilla beans | Lindt |
| Day 2/S | Chocolate Milk | Reduced Fat Milk, Sugar, Cocoa (Processed with Alkali), Cocoa, Salt, Carrageenan, Mono and Diglycerides, Vanillin (Artificial Flavor), Vitamin A Palmitate, Vitamin D3 | Cass Clay |
| Day 2/S | Canned Tomatoes | Tomatoes, tomato juice, sugar, less than 2% of : dried onions, salt, citric acid, dried celery, dried green bell pepper, calcium chloride, black pepper, garlic powder, dried oregano. | Campbell’s |
| Day 2/S | Condensed Tomato Soup | Tomato puree (water, tomato paste), wheat flour, sugar, water, contains less than 2% of: salt, citric acid, ascorbic acid (vitamin c), flavoring, celery extract, garlic oil. Contains: wheat | Campbell’s |
| Day 3/B | White Bread | Enriched Unbleached Flour (Wheat Flour, Malted Barley Flour, Niacin, Reduced Iron, Thiamine Mononitrate, Riboflavin, Folic Acid), Water, Bleached Oat Fiber, Sugar, Yeast, Wheat Gluten, Contains Less Than 2% Of Each Of The Following: White Rye Flour, Calcium Sulfate, Soybean Oil, Salt, Calcium Carbonate, Sodium Stearoyl Lactylate, Calcium Propionate (Preservative), Monoglycerides, Monocalcium Phosphate, Enzymes, Ascorbic Acid As A Dough Conditioner. | Country Hearth |
| Day 3/L | Chili Beans | Prepared Red Beans, Water, Contains Less Than 2% of: Modified Corn Starch, Salt, Tomato Paste, Spices, Sugar, Corn Oil, Garlic Powder, Onion Powder, Calcium Chloride (Firming Agent), Red Pepper. | Bush’s Best |
| Day 3/L | Salsa | Diced Tomatoes In Tomato Juice, Crushed Tomatoes (Water, Crushed Tomato Concentrate), Water, Jalapeno Peppers, Onions, Garlic, Distilled Vinegar, Dehydrated Onions, Salt, Cilantro, Natural Flavoring. | Pace |
| Day 3/L | Cornbread Mix | Wheat Flour, Degerminated Yellow Corn Meal, Sugar, Animal Shortening (Lard, Hydrogenated Lard, Tocopherols Preservative, BHT Preservative, Citric Acid Preservative), Contains Less than 2% of Each of the Following: Baking Soda, Sodium Acid Pyrophosphate, Monocalcium Phosphate, Salt, Wheat Starch, Niacin, Reduced Iron, Tricalcium Phosphate, Thiamine Mononitrate, Riboflavin, Folic Acid, Silicon Dioxide. | Jiffy |
| Day 3/L | Shortbread Cookies | Enriched flour (wheat flour, niacin, reduced iron, vitamin B1 [thiamin mononitrate], vitamin B2 [riboflavin], folic acid), soybean and palm oil, sugar, cashews.Contains 2% or less of salt, natural and artificial flavors, eggs, baking soda, whey protein concentrate, soy lecithin. | Keebler |
| Day 3/S | Beefy Mushroom Soup | Beef stock (water, dried beef stock), water, seasoned beef, beef broth and modified cornstarch product (beef, beef broth, salt, modified cornstarch, sodium phosphate, black pepper), mushrooms, modified cornstarch, contains less than 2% of: salt, wheat flour, beef stock, vegetable oil, yeast extract, monosodium glutamate, dried onions, caramel color, natural flavoring, hydrolyzed wheat gluten, hydrolyzed soy protein, beef tallow, dried beef. Contains: wheat, soy | Campbell’s |
| Day 4/B | White Bread | Enriched unbleached flour (wheat flour, malted barley flour, niacin, ferrous sulfate, thiamine hydrochloride, riboflavin, folic acid), water, high fructose corn syrup, yeast, liquid soybean oil. Contains less than 2% of each of the following: salt, dough conditioners (monoglycerides, sodium stearoyl lactylate, ascorbic acid, azodicarbonamide, calcium sulfate, calcium peroxide, enzymes), monocalcium phosphate, calcium propionate (preservative). | Lakeland |
| Day 4/L | Pepperoni | Pork, Beef, Salt, Contains 2% or Less of Water, Dextrose, Spices, Lactic Acid Starter Culture, Oleoresin of Paprika, Garlic Powder, Sodium Nitrite, BHA, BHT, Citric Acid. | Hormel |
| Day 4/L | Pizza Sauce | Tomato Puree (Water, Tomato Paste), Corn Syrup, Salt, Modified Corn Starch, Soybean Oil, Onion Powder, Natural Flavor, Carrot Fiber, Spices, Citric Acid, Garlic Powder, Potassium Sorbate (Preservative). | Contadina |
| Day 4/L | Mozzarella | Low-Moisture Part-Skim Mozzarella Cheese (Cultured Pasteurized Reduced Fat Milk, Salt, Enzymes), Anticake (Potato Starch, Powdered Cellulose), Natamycin (Natural Mold Inhibitor). | Essential Everyday |
| Day 4/L | Breadsticks | **Breadstick:** Enriched Wheat Flour (Wheat Flour, Malted Barley Flour, Niacin, Iron, Thiamine Mononitrate, Riboflavin, Folic Acid), Water, Sugar, Soybean Oil, Contains 2% or Less of: Salt, Yeast, Dough Conditioners [Datem, Calcium Sulfate, Enzymes, Ascorbic Acid, L-Cysteine], Corn Grits, Malted Barley, Mono and Diglycerides, Dextrose, Citric Acid, Cornmeal. **Spread:** Soybean Oil, Water, Palm Oil, Parmesan Cheese (Part-Skim Milk, Cheese Culture, Salt, Enzymes), Garlic*, Salt, Mono and Diglycerides, Parsley*, Nonfat Dry Milk, Whey, Beta Carotene Added for Color .*Dehydrated | New York Bakery |
| Day 4/S | Rice Krispie | Toasted rice cereal (rice, sugar, salt, malt flavor), corn syrup, fructose, marshmallows (corn syrup, sugar, modified food starch, gelatin), vegetable oil (soybean and palm oil with tbhq for freshness), sugar, corn syrup solids. contains 2% or less of vegetable glycerin, dextrose, gelatin, natural and artificial flavors (contains milk), salt, datem, acetylated monoglycerides, soy lecithin, bht for freshness. | Kellogg’s |
| Day 5/B | Frosted Flakes Cereal | Milled corn, sugar, malt flavor, contains 2% or less of salt. vitamins and minerals: iron (ferric phosphate), niacinamide, vitamin b6 (pyridoxine hydrochloride), vitamin b2 (riboflavin), vitamin b1 (thiamin hydrochloride), folic acid, vitamin d3, vitamin b12. | Kellogg’s |
| Day 5/L | Mustard | Distilled White Vinegar, Mustard Seed, Water, Salt, Turmeric, Natural Flavor and Spices. | Heinz |
| Day 5/L | Cheetos | Enriched Corn Meal (Corn Meal, Ferrous Sulfate, Niacin, Thiamin Mononitrate, Riboflavin, Folic Acid), Vegetable Oil (Corn, Canola, and/or Sunflower Oil), Cheese Seasoning (Whey, Cheddar Cheese [Milk, Cheese Cultures, Salt, Enzymes], Canola Oil, Maltodextrin [Made from Corn], Natural and Artificial Flavors, Salt, Whey Protein Concentrate, Monosodium Glutamate, Lactic Acid, Citric Acid, Artificial Color [Yellow 6]), and Salt. | Frito Lay |
| Day 5/S | Cream of Mushroom Soup | Water, Mushrooms, Vegetable Oil (Corn, Canola, And/Or Soybean), Modified Cornstarch, Wheat Flour, Salt, Cream, Whey, Soy Protein Concentrate, Monosodium Glutamate, Yeast Extract, Dried Garlic, Natural Flavoring. Contains: Wheat, Milk, Soy | Campbell’s |

# Supplemental Table 5. Ultra-Processed Foods included in Less-Processed Western Menu

| **Day/Meal** | **Food Description** | **Ingredients** |
| --- | --- | --- |
| Day 1/S | Homemade Stuffing* | Homemade white bread (instant yeast, flour, sugar, water, milk, butter), yellow onion, celery, thyme leaves, ground sage, rosemary, salt, pepper, chicken/veggie broth |
| Day 1/S | Gravy | Water, Organic Coconut Cream, Organic Turkey Bone Broth, Organic Tapioca Starch, Organic Coconut Aminos (Organic Coconut Blossom Nectar, Water, Himalayan Salt), Organic Chicken Fat, Organic Nutritional Yeast, Sea Salt, Organic Onion Powder, Poultry Spice Blend (Thyme, Sage, Marjoram, Rosemary, Black Pepper, Nutmeg), Organic Garlic Powder, Organic Turmeric. |
| Day 2/B | Bacon, Uncured | Pork, water, sea salt, Less than 2%: cane sugar, celery powder |
| Day 2/B | Pancake Mix, Dry | Organic Wheat Flour, Organic Cane Sugar, Organic Expeller-Pressed Sunflower Oil, Baking Powder (baking soda, sodium acid pyrophosphate, monocalcium phosphate), Sea Salt |
| Day 2/L | Breaded Chicken Tenders, Frozen | Organic White Meat Chicken (Chicken Never Administered Antibiotics or Fed Animal By-Products), Organic Wheat Flour, Water, Contains Less than 2% of the Following: Sea Salt, Organic Cane Sugar, Organic Paprika, Yeast, Organic Garlic Powder, Organic White Pepper, Breading Set in Organic Expeller Pressed Canola Oil. |
| Day 2/L | Mayonnaise | Organic Chosen Blend Oil (Organic High Oleic Safflower Oil, Organic Avocado Oil, Organic Coconut Oil), Filtered Water, Organic Egg Yolks, Organic Whole Eggs, Organic Distilled White Vinegar, Organic Mustard (Distilled Organic Vinegar, Water, Organic Mustard Seed, Salt, Organic Spices), Salt, Organic Rosemary Extract. |
| Day 2/L | Chocolate Bar with Coconut and Toffee | Organic Raw Cane Sugar (Fair Trade Certified by Fair Trade USA, Total 89.7% Fair Trade Ingredients), Organic Cacao Beans (Fair Trade Certified by Fair Trade USA, Total 89.7% Fair Trade Ingredients), Coconut Toffee (Organic Raw Cane Sugar (Fair Trade Certified by Fair Trade USA, Total 89.7% Fair Trade Ingredients), Organic Dessicated Coconut (Fair Trade Certified by Fair Trade USA, Total 89.7% Fair Trade Ingredients), Organic Butter, Sea Salt), Organic Cocoa Butter (Fair Trade Certified by Fair Trade USA, Total 89.7% Fair Trade Ingredients), Organic Whole Milk, Organic Butterfat. |
| Day 3/L | Cornbread Mix | Whole Grain Cornmeal, Whole Wheat Flour, Sugar, Buttermilk Powder, Salt, Baking Powder (Sodium Acid Pyrophosphate, Sodium Bicarbonate, Cornstarch, Monocalcium Phosphate), Baking Soda. |
| Day 3/L | Mexican Wedding Cookies | Grain Free Flour Blend (Almond Flour, Tapioca Flour, Arrowroot Flour, Cassava Flour, Chickpea Flour), Coconut Oil, Coconut Sugar, Powdered Sugar (Sugar, Tapioca Flour), Pecan Pieces, Avocado Oil, Apple Cider Vinegar, Sea Salt, Baking Soda, Cinnamon, Orange Peel Powder, Vanilla Extract, Jasmine Tea Extract |
| Day 3/D | Cream of Mushroom Soup | Filtered Water, Organic Mushrooms, Organic Onions, Organic Unbleached Wheat Flour, Organic High Oleic Safflower and/or Sunflower Oil, Organic Leeks, Organic Butter (Organic Cream, Salt), Organic cream, Organic Spices, Sea Salt, Organic Bay Leaves, Organic Black Pepper. |
| Day 4/L | Turkey Pepperoni | Turkey (Turkey Raised on Vegetarian Feed, on Family Farms, with at Least 33% More Space than Industry Standard and Environmental Enrichments to Promote Natural Behaviors and Well-Being), Sea Salt, Contains Less than 2% of the Following: Turbinado Sugar, Water, Spices, Cultured Celery Powder, Granulated Garlic, Cherry Powder, Lactic Acid Starter Culture (Not from Milk), Rosemary Extract, Oleoresin of Paprika, Lime Juice Concentrate. |
| Day 5/B | Brown Sugar Maple Instant Oatmeal | Gluten Free Whole Grain Rolled Oats*, Brown Sugar*, Cane Sugar*, Maple Sugar*, Rolled Amaranth*, Rolled Quinoa*, Sorghum Flour*, Natural Maple Flavor, Sea Salt. *Organic. |
| Day 5/L | Salty Snack Puffs | Organic Cassava Flour, Organic Coconut Oil, Organic Tapioca Starch, Organic Coconut Flour, Organic Sweet Potato Flour, Himalayan Salt. |

# Supplemental Table 6. Adjustments to Ultra-Processed Foods on Less-Processed Western (LPW) Menu to Match Ultra-Processed Foods on More-Processed Western (MPW) Menu

| LPW Food Description | MPW Day/Meal | Replacement Food from MPW | Ingredients in Replacement Food from MPW |
| --- | --- | --- | --- |
| Homemade Stuffing* | Day 1/S | Stuffing Mix | enriched wheat flour (wheat flour, niacin, reduced iron, thiamin mononitrate [vitamin b1], riboflavin [vitamin b2], folic acid), high fructose corn syrup, onion*, contains less than 2% of salt, hydrolyzed soy protein, yeast, interesterified soybean oil, cooked chicken*, celery*, monosodium glutamate, parsley*, spice, maltodextrin, potassium chloride, sugar, chicken broth*, turmeric (color), silicon dioxide as an anticaking agent, disodium guanylate, disodium inosinate, natural flavor, with bha, bht and rosemary extract as preservatives.*dried |
| Gravy | Day 1/S | Gravy | Chicken Broth, Modified Food Starch, Chicken Fat, Contains Less than 2% of Bleached Wheat Flour, Salt, Yeast Extract, Canola Oil, Whey (from Milk), Natural Flavor, Onion Powder, Black Pepper, Spices, Dextrose, Fruit Juice (Color) Turmeric (Color), Oleoresin Paprika (Color). |
| Bacon, Uncured | Day 2/B | Bacon | Cured with Water, Salt, Sugar, Sodium Erythorbate, Sodium Nitrite. |
| Pancake Mix, Dry | Day 2/B | Pancakes | Enriched flour (wheat flour, malted barley flour, niacin, reduced iron, vitamin B1 [thiamin mononitrate], vitamin B2 [riboflavin], folic acid), water, high fructose corn syrup, vegetable oil (soybean and/or canola), buttermilk, contains 2% or less of leavening (baking soda, sodium aluminum phosphate, monocalcium phosphate), salt, soy lecithin, eggs. Vitamins and Minerals: Iron, vitamin A palmitate, vitamin B6 (pyridoxine hydrochloride), vitamin B12. |
| Breaded Chicken Tenders, Frozen | Day 2/L | Chicken Patty | Chicken patties (chicken, water, salt, autolyzed yeast extract, sodium phosphates, natural flavorings), breader (bleached wheat flour, salt, dextrose, spice, soybean oil, yeast, extractives of paprika), batter (water, yellow corn flour, corn starch, salt, spices, guar gum, leavening [sodium acid pyrophosphate, sodium bicarbonate, monocalcium phosphate], garlic powder). Fried in vegetable oil. |
| Mayonnaise | Day 2/L | Miracle Whip | Water, soybean oil, high fructose corn syrup, vinegar, modified cornstarch, eggs, salt, natural flavor, mustard flour, potassium sorbates A preservative, paprika, spice, dried garlic. Contains: egg. |
| Chocolate Bar with Coconut and Toffee | Day 2/L | Dark Chocolate | Chocolate, sugar, cocoa butter, soy lecithin (emulsifier), bourbon vanilla beans |
| Cornbread Mix | Day 3/L | Cornbread Mix | Wheat Flour, Degerminated Yellow Corn Meal, Sugar, Animal Shortening (Lard, Hydrogenated Lard, Tocopherols Preservative, BHT Preservative, Citric Acid Preservative), Contains Less than 2% of Each of the Following: Baking Soda, Sodium Acid Pyrophosphate, Monocalcium Phosphate, Salt, Wheat Starch, Niacin, Reduced Iron, Tricalcium Phosphate, Thiamine Mononitrate, Riboflavin, Folic Acid, Silicon Dioxide. |
| Mexican Wedding Cookies | Day 3/L and Day 5/D | Shortbread Cookies | Enriched flour (wheat flour, niacin, reduced iron, vitamin B1 [thiamin mononitrate], vitamin B2 [riboflavin], folic acid), soybean and palm oil, sugar, cashews.Contains 2% or less of salt, natural and artificial flavors, eggs, baking soda, whey protein concentrate, soy lecithin. |
| Cream of Mushroom Soup | Day 5/S | Cream of Mushroom Soup | Water, Mushrooms, Vegetable Oil (Corn, Canola, And/Or Soybean), Modified Cornstarch, Wheat Flour, Salt, Cream, Whey, Soy Protein Concentrate, Monosodium Glutamate, Yeast Extract, Dried Garlic, Natural Flavoring. Contains: Wheat, Milk, Soy |
| Turkey Pepperoni | Day 4/L | Pepperoni | Pork, Beef, Salt, Contains 2% or Less of Water, Dextrose, Spices, Lactic Acid Starter Culture, Oleoresin of Paprika, Garlic Powder, Sodium Nitrite, BHA, BHT, Citric Acid. |
| Brown Sugar Maple Instant Oatmeal | Day 5/B | Frosted Corn Cereal | Milled corn, sugar, malt flavor, contains 2% or less of salt. vitamins and minerals: iron (ferric phosphate), niacinamide, vitamin b6 (pyridoxine hydrochloride), vitamin b2 (riboflavin), vitamin b1 (thiamin hydrochloride), folic acid, vitamin d3, vitamin b12. |
| Salty Snack Puffs | Day 5/L | Cheesy Corn Snacks | Enriched Corn Meal (Corn Meal, Ferrous Sulfate, Niacin, Thiamin Mononitrate, Riboflavin, Folic Acid), Vegetable Oil (Corn, Canola, and/or Sunflower Oil), Cheese Seasoning (Whey, Cheddar Cheese [Milk, Cheese Cultures, Salt, Enzymes], Canola Oil, Maltodextrin [Made from Corn], Natural and Artificial Flavors, Salt, Whey Protein Concentrate, Monosodium Glutamate, Lactic Acid, Citric Acid, Artificial Color [Yellow 6]), and Salt. |

# Supplemental Table 7. Adjusted Less-Processed Western Menu

| **DAY 1 BREAKFAST** | **DAY 2 BREAKFAST** | **DAY 3 BREAKFAST** | **DAY 4 BREAKFAST** | **DAY 5 BREAKFAST** |
| --- | --- | --- | --- | --- |
| Cranberry Juice  Prosciutto Egg Cups  Homemade White Bread  Salted Butter  Berry jam without pectin | Grape Juice  ^*^Pancakes  Salted Butter  Pure Maple Syrup ^*^Bacon | Vanilla Steamer  Scrambled Eggs  Homemade White Bread  Salted Butter  Berry jam without pectin | Apple Juice  Ricotta Stuffed French Toast  Salted Butter  Maple Syrup  ^*^Bacon | Grape Juice  Almond Steamer  Simple Syrup  ^*^Frosted Corn Flakes  ^*^Whole Milk  Homemade White Bread  Sunflower Butter  Honey |
| **DAY 1 LUNCH** | **DAY 2 LUNCH** | **DAY 3 LUNCH** | **DAY 4 LUNCH** | **DAY 5 LUNCH** |
| Fruit Punch  Taco Salad  Taco Meat  Iceberg Lettuce  Grape Tomatoes  Shredded pepperjack  Sour Cream  Mild Salsa  White Corn Tortilla Chips | Lemonade  Chicken Sandwich  ^*^Breaded Chicken Patty  Homemade Hamburger Buns  ^*^Miracle Whip  Iceberg Lettuce  White Corn Tortilla Chips  Salted Cashews  ^*^Dark Chocolate | Whole Milk  Chili  Slow Cooker Beans  Ground Beef  Celery  Mild Salsa  Sunflower Seeds  ^*^Cornbread  Honey  ^*^Shortbread Cookies | Fruit Punch  Pizza Hotdish  Spiral Macaroni  Ground Beef  ^*^Pepperoni  Pizza Sauce  Shredded Mozzarella  Homemade Breadsticks  Salted Whole Cashews  ^*^Chocolate Cookies | Lemonade  Ham Sandwich  Homemade Hamburger Buns  Mustard  Ham  Iceberg Lettuce  Macaroni Salad  Elbow Macaroni  Carrots  Celery  Peas  Green Onions  Mayonnaise  Mustard  ^*^Chips  ^*^Dark Chocolate |
| **DAY 1 DINNER** | **DAY 2 DINNER** | **DAY 3 DINNER** | **DAY 4 DINNER** | **DAY 5 DINNER** |
| Lemonade  Chicken & Stuffing  ^[[1]](#footnote-1)^Stuffing  Salted Butter  Sunflower Seeds  Chicken  ^*^Gravy  Green Beans  Homemade Dinner Rolls  Salted Butter  Homemade Brownie | Whole Milk  Hamburger Hotdish  Elbow Macaroni  Ground Beef  Tomatoes  Cream of Tomato Soup  Corn  Homemade Dinner Rolls  Salted Butter  Homemade Brownie | Fruit Punch  Pork & Noodles  Egg Noodles  Pork Tenderloin  Sour Cream  ^*^Beefy Mushroom Soup  Peas  Steamed Carrots  Homemade Brownie | Chicken & Potatoes  Red Potatoes  Chicken  ^*^Gravy  Chicken Seasoning  Peas  Homemade Dinner Rolls  Salted Butter  Homemade Crispy Rice Treats | Whole Milk  Tator Tot Hotdish  Ground Beef  Corn  ^*^Cream of Mushroom Soup  Onion Powder  Garlic Powder  Homemade tater tots  Homemade dinner rolls  Salted Butter  ^*^Shortbread Cookies |

# Supplemental Table 8. Cost Estimations for the Less Processed Western Diet

|  | **Total product weight (g)** | **Menu weight (g)** | **Unit Amount** | **Unit Issue** | **Unit Price ($)** | **Price in Menu ($)** | **Total Price ($)** |
| --- | --- | --- | --- | --- | --- | --- | --- |
| **FRUIT GROUP** | | | | | | | |
| Fresh, Lemons | 324 | 241 | 3 | each | $0.99 | $2.21 | $2.97 |
| Frozen, Berry Medley, Bag | 1130 | 86 | 1 | bag | $14.19 | $1.08 | $14.19 |
| Refrigerated, Juice, Orange, No Pulp, Original, Bottle | 1623 | 96 | 1 | bottle | $5.49 | $0.32 | $5.49 |
| Shelf Stable, Juice, Apple, 100% Juice, Bottle | 1996 | 276 | 1 | bottle | $4.49 | $0.62 | $4.49 |
| Shelf Stable, Juice, Concord Grape, Organic, Bottle | 1012.7 | 456 | 1 | bottle | $7.75 | $3.49 | $7.75 |
| Shelf Stable, Juice, Cranberry, Pure, Unsweetened, Bottle | 1000 | 180 | 1 | bottle | $5.38 | $0.97 | $5.38 |
| **VEGETABLE GROUP** | | | | | | | |
| Fresh, Carrots, Baby-Cut, Bag | 453 | 50 | 1 | bag | $1.69 | $0.19 | $1.69 |
| Fresh, Celery, Bag | 512 | 39 | 1 | bag | $1.99 | $0.15 | $1.99 |
| Fresh, Lettuce, Iceberg, Shredded, Bag | 226 | 100 | 1 | bag | $2.49 | $1.10 | $2.49 |
| Fresh, Onions, Green, Bunch | 105 | 10 | 1 | bunch | $0.99 | $0.09 | $0.99 |
| Fresh, Onions, Yellow [*1 med ≈ 0.63 lb/10 oz/283 g; amt adj for 1 med*] | 453 | 12 | 0.3 | lb | $1.88 | $0.05 | $0.56 |
| Fresh, Potatoes, Red, Bag | 2270 | 60 | 1 | bag | $3.49 | $0.09 | $3.49 |
| Fresh, Potatoes, Russet, Bag | 2270 | 36 | 1 | bag | $3.99 | $0.06 | $3.99 |
| Fresh, Spinach, Bag | 212 | 60 | 1 | pkg | $3.49 | $0.99 | $3.49 |
| Fresh, Tomatoes, Grape | 283.5 | 40 | 1 | pkg | $3.99 | $0.56 | $3.99 |
| Fresh, Tomatoes | 453 | 60 | 0.5 | lb | $2.79 | $0.37 | $1.40 |
| Frozen, Corn, Baby Gold & White, Bag | 408 | 80 | 1 | bag | $3.49 | $0.68 | $3.49 |
| Frozen, Green Beans, Cut, Steamfresh, Bag | 283.5 | 40 | 1 | bag | $2.49 | $0.35 | $2.49 |
| Frozen, Peas, Green, Bag | 907 | 80 | 1 | bag | $4.79 | $0.42 | $4.79 |
| Shelf Stable, Pizza Sauce, Jar | 360 | 100 | 1 | jar | $4.78 | $1.33 | $4.78 |
| Shelf Stable, Salsa, Thick and Chunky, Jar | 284 | 122 | 1 | bottle | $7.09 | $3.05 | $7.09 |
| Shelf Stable, Soup, Cream of Tomato, Can | 411 | 60 | 1 | can | $4.29 | $0.63 | $4.29 |
| Shelf Stable, Soup, Cream of Mushroom, Can | 400 | 100 | 1 | can | $4.29 | $1.07 | $4.29 |
| **FATS & OILS** | | | | | | | |
| Refrigerated, Butter, Salted, Sticks, Box | 1120 | 145 | 1 | box | $5.85 | $3.79 | $5.85 |
| Shelf Stable, Cooking Spray, Canola Oil, Non-Stick, Can | 170 | 5 | 1 | can | $3.79 | $0.11 | $3.79 |
| Shelf Stable, Gravy, Classic, Jar | 340 | 120 | 1 | jar | $9.99 | $3.53 | $9.99 |
| Shelf Stable, Mayonnaise, Jar | 346 | 45 | 1 | jar | $8.59 | $1.12 | $8.59 |
| Shelf Stable, Olive Oil, Bottle | 447 | 14 | 1 | bottle | $9.29 | $0.29 | $9.29 |
| **MILK, YOGURT, EGGS, & CHEESE GROUP** | | | | | | | |
| Refrigerated, Cheese, Cheddar, White Extra Sharp, Block | 198 | 20 | 1 | pkg | $3.89 | $0.39 | $3.89 |
| Refrigerated, Cheese, Mozzarella, Shredded | 226 | 20 | 1 | pkg | $3.39 | $0.30 | $3.39 |
| Refrigerated, Cheese, Pepper Jack, Block | 198 | 20 | 1 | pkg | $3.89 | $0.39 | $3.89 |
| Refrigerated, Cheese, Ricotta, Authentic Italian Flavor, Tub | 425 | 20.5 | 1 | tub | $7.55 | $0.36 | $7.55 |
| Refrigerated, Cream, Heavy Whipping, Premium, Ultra-Pasteurized, Carton | 250 | 2.5 | 1 | carton | $5.99 | $0.06 | $5.99 |
| Refrigerated, Eggs, Whole, Fresh, Grade A Large | 1200 | 350 | 1 | carton | $3.78 | $2.21 | $3.78 |
| Refrigerated, Half & Half, Premium, Ultra-Pasteurized, Carton | 484 | 11 | 1 | carton | $2.39 | $0.05 | $2.39 |
| Refrigerated, Milk, Whole, Vitamin D, Ultra Pasteurized, Grade A, Carton | 2000 | 1181 | 1 | carton | $5.79 | $3.42 | $5.79 |
| Refrigerated, Sour Cream, Pure & Natural, Tub | 453 | 40 | 1 | tub | $3.89 | $0.34 | $3.89 |
| Shelf Stable, Cheese, Parmesan, Grated, Bottle | 225 | 0.42 | 1 | bottle | $3.99 | $0.01 | $3.99 |
| **MEAT, POULTRY, FISH, & NUTS GROUP** | | | | | | | |
| Frozen, Chicken Strips, Formed & Breaded, White Meat Chicken, Box | 226 | 65 | 1 | pkg | $10.29 | $2.96 | $10.29 |
| Refrigerated, Bacon, Uncured, Sunday, Hickory Smoked (*adjusted for yield*) | 226 | 40 | 1 | pkg | $7.19 | $1.27 | $7.19 |
| Refrigerated, Beef, Ground, Lean, 93/7 | 453 | 400 | 1 | lbs | $6.15 | $5.43 | $6.15 |
| Refrigerated, Chicken, Breast Fillets, Boneless, Skinless | 453 | 160 | 1 | pkg | $7.09 | $2.50 | $7.09 |
| Refrigerated, Ham, Black Forest, Uncured, Deli, Sliced | 198 | 60 | 1 | pkg | $8.35 | $2.53 | $8.35 |
| Refrigerated, Ham, Prosciutto, Dry Cured, Sliced | 170 | 38 | 1 | pkg | $7.78 | $1.74 | $7.78 |
| Refrigerated, Pork, Half Loin | 453 | 80 | 1 | pkg | $2.99 | $0.53 | $2.99 |
| Shelf Stable, Cashews, Whole, Salted, Deluxe, Can | 240 | 50 | 1 | can | $8.59 | $1.79 | $8.59 |
| Shelf Stable, Peanut Butter, Natural, Creamy, Jar | 453 | 15 | 1 | jar | $4.49 | $0.15 | $4.49 |
| Shelf Stable, Pinto Beans, Dried, Bag | 907 | 15 | 1 | bag | $3.59 | $0.06 | $3.59 |
| Shelf Stable, Sunflower Butter, Natural, Jar | 453 | 15 | 1 | jar | $6.99 | $0.23 | $6.99 |
| Shelf Stable, Sunflower Seeds, Roasted & Salted, Original, Bag (*adj for yield*) | 163 | 30 | 1 | bag | $2.39 | $0.44 | $2.39 |
| Shelf Stable, Turkey Pepperoni, Uncured, Resealable Bag | 120 | 10 | 1 | pkg | $6.89 | $0.57 | $6.89 |
| **SEASONINGS & MISCELLANEOUS** | | | | | | | |
| Fresh, Parsley | 74 | 1.5 | 1 | bunch | $1.99 | $0.04 | $1.99 |
| Shelf Stable, Broth, Chicken, Organic, Carton | 907 | 76 | 1 | carton | $4.59 | $0.38 | $4.59 |
| Shelf Stable, Mustard, Original Yellow, Bottle | 340 | 8 | 1 | bottle | $2.29 | $0.05 | $2.29 |
| Shelf Stable, Seasoning, Black Pepper, Ground, Pure, Can | 85 | 1 | 1 | can | $5.99 | $0.07 | $5.99 |
| Shelf Stable, Seasoning, Garlic Powder, Jar | 88 | 1.1 | 1 | jar | $3.69 | $0.05 | $3.69 |
| Shelf Stable, Seasoning, Italian Seasoning, Dried, Jar | 21 | 0.09 | 1 | jar | $2.09 | $0.01 | $2.09 |
| Shelf Stable, Seasoning, Onion Powder, Jar | 74 | 1 | 1 | jar | $3.19 | $0.04 | $3.19 |
| Shelf Stable, Seasoning, Poultry Seasoning, Jar | 18 | 2 | 1 | jar | $3.79 | $0.42 | $3.79 |
| Shelf Stable, Seasoning, Taco Seasoning, Mild, Paleo, Preservative Free | 37 | 3 | 1 | pkg | $2.48 | $0.20 | $2.48 |
| **SWEETS/SNACK GROUP** | | | | | | | |
| Shelf Stable, Candy, Dark Chocolate, Coconut Toffee, Organic, Bar | 80 | 50 | 1 | each | $4.45 | $2.78 | $4.45 |
| Shelf Stable, Candy, Mini Chips, 100% Real Chocolate, Semi-Sweet, Bag | 283 | 6.2 | 1 | bag | $7.05 | $0.15 | $7.05 |
| Shelf Stable, Chips, Tortilla, White Corn, Bag | 311.8 | 65 | 1 | bag | $2.69 | $0.56 | $2.69 |
| Shelf Stable, Cookies, Mexican Wedding, Grain Free, Bag | 127.8 | 90 | 1 | bag | $5.29 | $3.73 | $5.29 |
| Shelf Stable, Honey, Clover, US Grade A, No Drip Cap, Bottle | 340 | 60 | 1 | bottle | $4.39 | $0.77 | $4.39 |
| Shelf Stable, Puffs, Paleo, Himalayan Pink Salt, Grain Free, Bag | 142 | 28 | 1 | bag | $3.78 | $0.75 | $3.78 |
| Shelf Stable, Syrup, Maple, 100% Pure, Bottle | 333 | 151.7 | 1 | bottle | $11.25 | $5.13 | $11.25 |
| **BREAD, CEREAL, GRAINS & PASTA GROUP** | | | | | | | |
| Shelf Stable, Cereal, Puffed Rice, Natural, Box | 170 | 4.7 | 1 | box | $2.99 | $0.08 | $2.99 |
| Shelf Stable, Cornbread & Muffin Mix, Stone Ground, Bag | 680 | 55 | 1 | bag | $4.45 | $0.36 | $4.45 |
| Shelf Stable, Egg Noodles, Extra Wide, Dry, Bag | 453 | 100 | 1 | bag | $3.29 | $0.73 | $3.29 |
| Shelf Stable, Macaroni, Elbow, Dry, Box | 453 | 140 | 1 | box | $1.79 | $0.55 | $1.79 |
| Shelf Stable, Macaroni, Rotini, Dry, Box | 453 | 90 | 1 | box | $1.79 | $0.36 | $1.79 |
| Shelf Stable, Oatmeal, Instant, Brown Sugar Maple, Gluten Free, Box | 320 | 43 | 1 | box | $4.64 | $0.62 | $4.64 |
| Shelf Stable, Pancake & Waffle Mix, Almond Flour, Original, Box | 303 | 40 | 1 | box | $7.58 | $1.00 | $7.58 |
| **BAKERY/BAKED PRODUCT INGREDIENTS** | | | | | | | |
| Shelf Stable, Cocoa Powder, Unsweetened, Natural, Can | 226 | 13.44 | 1 | can | $5.09 | $0.30 | $5.09 |
| Shelf Stable, Extract, Almond, Pure, Bottle | 57 | 4.2 | 1 | bottle | $5.29 | $0.39 | $5.29 |
| Shelf Stable, Extract, Vanilla, Pure, Bottle | 29.4 | 10.5 | 1 | bottle | $4.89 | $1.75 | $4.89 |
| Shelf Stable, Flour, All Purpose, Enriched, Unbleached, Pre-sifted | 4530 | 402.93 | 1 | bag | $6.49 | $0.58 | $6.49 |
| Shelf Stable, Salt, Iodized, Table, Cylinder | 737 | 9.87 | 1 | each | $1.19 | $0.02 | $1.19 |
| Shelf Stable, Spice, Cinnamon, Ground, Jar | 67 | 1.41 | 1 | jar | $3.79 | $0.08 | $3.79 |
| Shelf Stable, Spice, Nutmeg, Ground, Jar | 31 | 0.09 | 1 | jar | $5.79 | $0.02 | $5.79 |
| Shelf Stable, Sugar, Light Brown, Bag | 907 | 27.5 | 1 | bag | $3.09 | $0.09 | $3.09 |
| Shelf Stable, Sugar, Powdered, Bag | 907 | 0.21 | 1 | bag | $3.59 | $0.00 | $3.59 |
| Shelf Stable, Sugar, White, Granulated, Beet Sugar | 1810 | 123.13 | 1 | bag | $3.09 | $0.21 | $3.09 |
| Shelf Stable, Yeast, Active Dry, 3-count Package | 21 | 2.25 | 1 | pkg | $1.89 | $0.20 | $1.89 |
| Shelf Stable, Yeast, Instant Dry, Quick Rise, Gluten Free, Jar | 113.4 | 7.66 | 1 | jar | $7.79 | $0.53 | $7.79 |
|  |  |  |  |  | **TOTAL =** | **$ 79.46** | **$ 407.92** |
|  |  |  |  |  | Avg/Day = | $ 15.89 | $ 81.58 |

# Supplemental Table 9. Cost Estimations for the More Processed Western Diet

|  | **Total product weight (g)** | **Menu weight (g)** | **Unit Amount** | **Unit Issue** | **Unit Price ($)** | **Price in Menu ($)** | **Total Price ($)** |
| --- | --- | --- | --- | --- | --- | --- | --- |
| **FRUIT GROUP** | | | | | | | |
| Refrigerated, Juice, Fruit Punch, Juice Drink, Plastic Bottle | 1542.86 | 540 | 1 | bottle | $2.99 | $1.05 | $2.99 |
| Refrigerated, Juice, Grape, Concord, Juice Cocktail, Carton | 1800 | 360 | 1 | carton | $3.79 | $0.76 | $3.79 |
| Refrigerated, Juice, Lemonade, All Natural, Bottle | 1565.22 | 360 | 1 | bottle | $2.99 | $0.69 | $2.99 |
| Refrigerated, Juice, Peach, Premium, Carton | 1800 | 360 | 1 | carton | $2.29 | $0.46 | $2.29 |
| Shelf Stable, Juice, Cranberry Raspberry, Juice Cocktail, USA Grown, Plastic Bottle | 1894.74 | 360 | 1 | bottle | $3.59 | $0.68 | $3.59 |
| **VEGETABLE GROUP** | | | | | | | |
| Fresh, Carrots, Sweet Petites, Premium, Bag | 340 | 50 | 1 | bag | $2.99 | $0.44 | $2.99 |
| Fresh, Celery, Green, Bag | 512 | 25 | 1 | bag | $1.99 | $0.10 | $1.99 |
| Fresh, Lettuce, Romaine, Hearts, Whole Leaves, Bag | 624 | 100 | 1 | bag | $3.99 | $0.64 | $3.99 |
| Fresh, Onions, Green | 105 | 20 | 1 | each | $0.99 | $0.19 | $0.99 |
| Fresh, Potatoes, Red, Bag | 2270 | 60 | 1 | bag | $3.49 | $0.09 | $3.49 |
| Fresh, Tomatoes, Grape | 283.5 | 40 | 1 | pkg | $2.99 | $0.42 | $2.99 |
| Frozen, Corn, Super Sweet, Bag | 453 | 80 | 1 | bag | $2.49 | $0.44 | $2.49 |
| Frozen, Green Beans, Cut, Bag | 453 | 40 | 1 | bag | $2.49 | $0.22 | $2.49 |
| Frozen, Peas, Bag | 453 | 75 | 1 | bag | $2.49 | $0.41 | $2.49 |
| Frozen, Potato Crowns, Seasoned Shredded Potatoes, Bag | 850 | 80 | 1 | bag | $3.89 | $0.37 | $3.89 |
| Shelf Stable, Beans, Chili, Red Beans, Mild Chili Sauce, Can | 453 | 80 | 1 | can | $1.89 | $0.33 | $1.89 |
| Shelf Stable, Salsa, Restaurant Style, Mild Recipe, Bottle | 453 | 120 | 1 | bottle | $3.19 | $0.85 | $3.19 |
| Shelf Stable, Sauce, Pizza, Pizza Squeeze, Plastic Squeeze Bottle | 425 | 90 | 1 | bottle | $2.09 | $0.44 | $2.09 |
| Shelf Stable, Soup, Beefy Mushroom, Condensed, Can | 297 | 40 | 1 | can | $1.99 | $0.27 | $1.99 |
| Shelf Stable, Soup, Cream of Mushroom, Condensed, Can | 304 | 30 | 1 | can | $1.89 | $0.19 | $1.89 |
| Shelf Stable, Soup, Tomato, Condensed, Can | 304 | 60 | 1 | can | $1.79 | $0.35 | $1.79 |
| Shelf Stable, Tomatoes, Diced, with Onion, Celery & Bell Pepper, Can | 410 | 60 | 1 | can | $1.49 | $0.22 | $1.49 |
| **FATS & OILS** | | | | | | | |
| Refrigerated, Butter, Unsalted, Sticks, Box | 453 | 63.5 | 1 | box | $5.85 | $0.82 | $5.85 |
| Shelf Stable, Cooking Spray, 100% Canola Oil, Original, Can [*est 1 sec spray used daily*] | 170 | 5 | 1 | can | $3.79 | $0.11 | $3.79 |
| Shelf Stable, Dressing, Miracle Whip, Original, jar | 409 | 45 | 1 | jar | $5.19 | $0.57 | $5.19 |
| Shelf Stable, Gravy, Classic Chicken, Homestyle, 12 oz Jar | 340 | 85 | 1 | jar | $2.99 | $0.75 | $2.99 |
| Shelf Stable, Oil, Pure Vegetable, Soybean, Bottle [*amt for brownies, ½ cup per 1 box*] | 450 | 5 | 1 | bottle | $3.69 | $0.04 | $3.69 |
| **MILK, YOGURT, EGGS, & CHEESE GROUP** | | | | | | | |
| Refrigerated, Cheese, Cheddar, Sharp, Classic Cut/Shredded, Bag | 226 | 50 | 1 | bag | $3.39 | $0.75 | $3.39 |
| Refrigerated, Cheese, Mozzarella, Low Moisture Part-Skim, Classic Cut/Shredded, Bag | 226 | 15 | 1 | bag | $3.39 | $0.23 | $3.39 |
| Refrigerated, Eggs, Whole, Fresh, Grade A Large | 600 | 168.75 | 1 | each | $3.78 | $1.06 | $3.78 |
| Refrigerated, Milk, Swiss Chocolate, 2% Reduced Fat, Vitamin A&D, Plastic Bottle | 1000 | 540 | 1 | qt | $2.49 | $1.34 | $2.49 |
| Refrigerated, Milk, White, 1% Low Fat, Vitamin A & D, Plastic Bottle | 1000 | 155.5 | 1 | qt | $2.29 | $0.36 | $2.29 |
| Refrigerated, Sour Cream, Dairy Pure, All Natural | 226 | 40 | 1 | tub | $1.29 | $0.23 | $1.29 |
| **MEAT, POULTRY, FISH, & NUTS GROUP** | | | | | | | |
| Frozen, Chicken, Breaded Chicken Patty, Made with Drum & Thigh Meat, Bag | 680 | 65 | 1 | bag | $6.79 | $0.65 | $6.79 |
| Refrigerated, Bacon, 100% Real Thick Cut, Fully Cooked, Original, Ready to Eat | 71 | 40 | 1 | box | $5.19 | $2.92 | $5.19 |
| Refrigerated, Beef, Ground, Lean, 93/7, 100% Pure, Raw, Chub | 453 | 400 | 1 | lbs | $6.15 | $5.43 | $6.15 |
| Refrigerated, Chicken, All Natural, Breast Fillets, Boneless, Skinless, Raw | 453 | 160 | 1 | pkg | $7.09 | $2.50 | $7.09 |
| Refrigerated, Ham, Deli Fresh, Black Forest, Water Added | 255 | 60 | 1 | pkg | $7.25 | $1.71 | $7.25 |
| Refrigerated, Ham, Mini Cubed, Fully Cooked, 96% Fat Free, Zipper Bag | 226 | 20 | 1 | pkg | $3.79 | $0.34 | $3.79 |
| Refrigerated, Pepperoni, Original, Pillow Pack, Resealable Zip-Pak | 170 | 15 | 1 | pkg | $5.15 | $0.45 | $5.15 |
| Refrigerated, Pork Loin, Boneless Pork Chop, THIN cut, Raw | 453 | 60 | 1 | lbs | $4.99 | $0.66 | $4.99 |
| Shelf Stable, Nuts, Cashews, Whole | 240 | 45 | 1 | can | $8.59 | $1.61 | $8.59 |
| Shelf Stable, Sunflower Butter, Creamy, Jar | 453 | 15 | 1 | jar | $6.99 | $0.23 | $6.99 |
| Shelf Stable, Sunflower Kernels, Dry Roasted, Pop & Pour, Jar | 165 | 30 | 1 | jar | $1.99 | $0.36 | $1.99 |
| **SEASONINGS & MISCELLANEOUS** | | | | | | | |
| Shelf Stable, Mustard, Yellow, 100% Natural, Squeeze, Plastic Bottle, 14 oz | 396 | 8 | 1 | bottle | $2.29 | $0.05 | $2.29 |
| Shelf Stable, Seasoning, Garlic Powder, Jar | 88 | 1 | 1 | jar | $3.69 | $0.04 | $3.69 |
| Shelf Stable, Seasoning, Montreal Chicken, Grill Mates, Jar | 77 | 2 | 1 | jar | $3.69 | $0.10 | $3.69 |
| Shelf Stable, Seasoning, Onion Powder, Jar | 74 | 1 | 1 | jar | $3.19 | $0.04 | $3.19 |
| Shelf Stable, Seasoning, Taco, Original, Dry, Envelope | 28 | 3 | 1 | pkg | $1.25 | $0.13 | $1.25 |
| **SWEETS/SNACK GROUP** | | | | | | | |
| Shelf Stable, Bar, Rice Krispies Treats, Homestyle, Original, Box | 176 | 35 | 1 | box | $3.69 | $0.73 | $3.69 |
| Shelf Stable, Brownie Mix, Chewy Fudge, Thick and Fudgy, Box | 520 | 100 | 1 | box | $2.79 | $0.54 | $2.79 |
| Shelf Stable, Candy, Dark Chocolate, 70% Cocoa, Excellence, Bar | 100 | 50 | 1 | each | $3.39 | $1.70 | $3.39 |
| Shelf Stable, Cheetos, Crunchy, 8.5 oz Bag | 240 | 25 | 1 | bag | $5.69 | $0.59 | $5.69 |
| Shelf Stable, Chips, Tortilla, Doritos, Nacho Cheese, Bag | 262.1 | 25 | 1 | bag | $5.99 | $0.57 | $5.99 |
| Shelf Stable, Chips, Tortilla, Tostitos, Original, Restaurant Style, Bag | 340 | 40 | 1 | bag | $5.99 | $0.70 | $5.99 |
| Shelf Stable, Cookies, Sandies, Cashew Shortbread | 317 | 60 | 1 | pkg | $4.09 | $0.77 | $4.09 |
| Shelf Stable, Cookies, Sandwich, Oreo, Original | 376 | 60 | 1 | pkg | $5.29 | $0.84 | $5.29 |
| Shelf Stable, Corn Muffin, Mix, Box | 240 | 35.37 | 1 | box | $0.89 | $0.13 | $0.89 |
| Shelf Stable, Honey, Clover, US Grade A, No Drip Cap, Bottle | 453 | 60 | 1 | bottle | $6.09 | $0.81 | $6.09 |
| Shelf Stable, Jelly, Strawberry, Jar | 510 | 40 | 1 | jar | $4.19 | $0.33 | $4.19 |
| Shelf Stable, Syrup, Original, No High Fructose Corn Syrup, Bottle | 706 | 60 | 1 | bottle | $4.79 | $0.41 | $4.79 |
| **BREAD, CEREAL, GRAINS & PASTA GROUP** | | | | | | | |
| Fresh, Bread, Hamburger Buns, White, Old Fashioned, Unsliced | 425 | 110 | 1 | pkg | $4.49 | $1.16 | $4.49 |
| Fresh, Bread, Rolls, Dinner, White, Pull Apart | 425 | 120 | 1 | pkg | $4.49 | $1.27 | $4.49 |
| Fresh, Bread, Texas Toast, White, Enriched, Sandwich Bread, Sliced | 793 | 70 | 1 | pkg | $4.79 | $0.42 | $4.79 |
| Fresh, Bread, White, Kid’s Choice, 4 x Calcium & 2 x Fiber, Sliced | 680 | 160 | 1 | pkg | $4.39 | $1.03 | $4.39 |
| Frozen, Bread, Bread Sticks, with Real Garlic | 284 | 30 | 1 | box | $3.59 | $0.38 | $3.59 |
| Frozen, Pancakes, Buttermilk, Eggo, Box | 420 | 75 | 1 | box | $3.49 | $0.62 | $3.49 |
| Shelf Stable, Cereal, Frosted Flakes, Dry, Box | 382 | 30 | 1 | box | $5.79 | $0.45 | $5.79 |
| Shelf Stable, Pasta, Cavatappi, Enriched, Box | 453 | 45 | 1 | box | $2.19 | $0.22 | $2.19 |
| Shelf Stable, Pasta, Egg Noodles, Extra Wide, Dry, Bag | 340 | 50 | 1 | bag | $2.99 | $0.44 | $2.99 |
| Shelf Stable, Pasta, Elbow Macaroni, Enriched, Box | 453 | 70 | 1 | box | $2.19 | $0.34 | $2.19 |
| Shelf Stable, Stuffing, Stove Top, Chicken, Box | 170 | 80 | 1 | box | $2.99 | $1.41 | $2.99 |
| **BAKERY/BAKED PRODUCT INGREDIENTS** | | | | | | | |
| Shelf Stable, Extract, Vanilla, Pure Madagascar Vanilla Beans, Bottle | 29 | 1.5 | 1 | bottle | $5.49 | $0.28 | $5.49 |
| Shelf Stable, Salt, Iodized, Cylinder | 737 | 0.125 | 1 | each | $1.19 | $0.00 | $1.19 |
| Shelf Stable, Spice, Cinnamon, Ground | 67 | 0.25 | 1 | jar | $3.79 | $0.01 | $3.79 |
| Shelf Stable, Sugar, Light Brown | 907 | 1.25 | 1 | bag | $3.59 | $0.00 | $3.59 |
|  |  |  |  |  | **TOTAL =** | **$ 49.26** | **$ 282.33** |
|  |  |  |  |  | Avg/Day = | $ 9.85 | $ 56.47 |

# Supplemental Table 10. Cost Estimations for the adjusted Less Processed Western Diet

|  | **Total product weight (g)** | **Menu weight (g)** | **Unit Amount** | **Unit Issue** | **Unit Price ($)** | **Price in Menu ($)** | **Total Price ($)** |
| --- | --- | --- | --- | --- | --- | --- | --- |
| Fresh, Lemons | 324 | 241 | 3 | each | $0.99 | $2.21 | $2.97 |
| Frozen, Berry Medley, Bag | 1130 | 86 | 1 | bag | $14.19 | $1.08 | $14.19 |
| Refrigerated, Juice, Orange, No Pulp, Original, Bottle | 1623 | 96 | 1 | bottle | $5.49 | $0.32 | $5.49 |
| Shelf Stable, Juice, Apple, 100% Juice, Bottle | 1996 | 276 | 1 | bottle | $4.49 | $0.62 | $4.49 |
| Shelf Stable, Juice, Concord Grape, Organic, Bottle | 1012.7 | 456 | 1 | bottle | $7.75 | $3.49 | $7.75 |
| Shelf Stable, Juice, Cranberry, Pure, Unsweetened, Bottle | 1000 | 180 | 1 | bottle | $5.38 | $0.97 | $5.38 |
| **VEGETABLE GROUP** | | | | | | | |
| Fresh, Carrots, Baby-Cut, Bag | 453 | 50 | 1 | bag | $1.69 | $0.19 | $1.69 |
| Fresh, Celery, Bag | 498.67 | 39 | 1 | bag | $1.99 | $0.15 | $1.99 |
| Fresh, Lettuce, Iceberg, Shredded, Bag | 226 | 100 | 1 | bag | $2.49 | $1.10 | $2.49 |
| Fresh, Onions, Green, Bunch | 105 | 10 | 1 | bunch | $0.99 | $0.09 | $0.99 |
| Fresh, Potatoes, Red, Bag | 453 | 0.00 | 0.3 | lb | $- | $- | $- |
| Fresh, Potatoes, Russet, Bag | 2270 | 60 | 1 | bag | $3.49 | $0.09 | $3.49 |
| Fresh, Spinach, Bag | 2270 | 36 | 1 | bag | $3.99 | $0.06 | $3.99 |
| Fresh, Tomatoes, Grape | 212 | 60 | 1 | pkg | $3.49 | $0.99 | $3.49 |
| Fresh, Tomatoes, Hot House [*1 med* ≈ *0.5 lb/8 oz/226 g; amt adj for 1 med*] | 283.5 | 40 | 1 | pkg | $3.99 | $0.56 | $3.99 |
| Frozen, Corn, Baby Gold & White, Bag | 453 | 60 | 0.5 | lb | $2.79 | $0.37 | $1.40 |
| Frozen, Green Beans, Cut, Steamfresh, Bag | 408 | 80 | 1 | bag | $3.49 | $0.68 | $3.49 |
| Frozen, Peas, Green, Bag | 283.5 | 40 | 1 | bag | $2.49 | $0.35 | $2.49 |
| Shelf Stable, Pizza Sauce, Jar | 907 | 80 | 1 | bag | $4.79 | $0.42 | $4.79 |
| Shelf Stable, Salsa, Thick and Chunky, Jar | 360 | 100 | 1 | jar | $4.78 | $1.33 | $4.78 |
| Shelf Stable, Soup, Cream of Tomato, Can | 284 | 122 | 1 | bottle | $7.09 | $3.05 | $7.09 |
| Shelf Stable, Soup, Beefy Mushroom, Condensed, Can | 411 | 60 | 1 | can | $4.29 | $0.63 | $4.29 |
| Shelf Stable, Soup, Cream of Mushroom, Condensed, Can | 304 | 100 | 1 | can | $1.89 | $0.62 | $1.89 |
| **FATS & OILS** | | | | | | | |
| Refrigerated, Butter, Salted, Sticks, Box | 1120 | 107.06 | 1 | box | $ 5.85 | $ 2.80 | $ 5.85 |
| Shelf Stable, Cooking Spray, Canola Oil, Non-Stick, Can | 170 | 5 | 1 | can | $ 3.79 | $ 0.11 | $ 3.79 |
| Shelf Stable, Gravy, Classic Chicken, Homestyle, 12 oz Jar | 340 | 120 | 1 | jar | $ 2.99 | $ 1.06 | $ 2.99 |
| Shelf Stable, Dressing, Miracle Whip, Original, jar | 409 | 45 | 1 | jar | $ 5.19 | $ 0.57 | $ 5.19 |
| Shelf Stable, Olive Oil, Bottle | 447 | 14 | 1 | bottle | $ 9.29 | $ 0.29 | $ 9.29 |
| **MILK, YOGURT, EGGS, & CHEESE GROUP** | | | | | | | |
| Refrigerated, Cheese, Cheddar, White Extra Sharp, Block | 198 | 20 | 1 | pkg | $3.89 | $0.39 | $3.89 |
| Refrigerated, Cheese, Mozzarella, Shredded | 226 | 20 | 1 | pkg | $3.39 | $0.30 | $3.39 |
| Refrigerated, Cheese, Pepper Jack, Block | 198 | 20 | 1 | pkg | $3.89 | $0.39 | $3.89 |
| Refrigerated, Cheese, Ricotta, Authentic Italian Flavor, Tub | 425 | 20.5 | 1 | tub | $7.55 | $0.36 | $7.55 |
| Refrigerated, Cream, Heavy Whipping, Premium, Ultra-Pasteurized, Carton | 250 | 2.5 | 1 | carton | $5.99 | $0.06 | $5.99 |
| Refrigerated, Eggs, Whole, Fresh, Grade A Large | 1200 | 323.05 | 1 | carton | $3.78 | $2.04 | $3.78 |
| Refrigerated, Half & Half, Premium, Ultra-Pasteurized, Carton | 484 | 11 | 1 | carton | $2.39 | $0.05 | $2.39 |
| Refrigerated, Milk, Whole | 2000 | 1294.75 | 1 | carton | $5.79 | $3.75 | $5.79 |
| Refrigerated, Sour Cream, Pure & Natural, Tub | 453 | 40 | 1 | tub | $3.89 | $0.34 | $3.89 |
| Shelf Stable, Cheese, Parmesan, Grated, Bottle | 225 | 0.42 | 1 | bottle | $3.99 | $0.01 | $3.99 |
| **MEAT, POULTRY, FISH, & NUTS GROUP** | | | | | | | |
| Frozen, Chicken, Breaded Chicken Patty, Made with Drum & Thigh Meat, Bag | 680 | 65 | 1 | bag | $6.79 | $0.65 | $6.79 |
| Refrigerated, Bacon, 100% Real Thick Cut, Fully Cooked, Original, Ready to Eat | 226 | 40 | 1 | box | $5.19 | $0.92 | $5.19 |
| Refrigerated, Beef, Ground, Lean, 93/7, 100% Pure, Raw, Chub | 453 | 400 | 1 | lbs | $6.15 | $5.43 | $6.15 |
| Refrigerated, Chicken, Breast Fillets, Boneless, Skinless | 453 | 160 | 1 | pkg | $7.09 | $2.50 | $7.09 |
| Refrigerated, Ham, Black Forest, Uncured, Deli, Sliced | 198 | 60 | 1 | pkg | $8.35 | $2.53 | $8.35 |
| Refrigerated, Ham, Prosciutto, Dry Cured, Sliced | 170 | 38 | 1 | pkg | $7.78 | $1.74 | $7.78 |
| Refrigerated, Pork, Half Loin | 453 | 80 | 1 | pkg | $2.99 | $0.53 | $2.99 |
| Shelf Stable, Cashews, Whole, Salted, Deluxe, Can | 240 | 50 | 1 | can | $8.59 | $1.79 | $8.59 |
| Shelf Stable, Peanut Butter, Natural, Creamy, Jar | 453 | 15 | 1 | jar | $4.49 | $0.15 | $4.49 |
| Shelf Stable, Pinto Beans, Dried, Bag | 907 | 15 | 1 | bag | $3.59 | $0.06 | $3.59 |
| Shelf Stable, Sunflower Butter, Natural, Jar | 453 | 15 | 1 | jar | $6.99 | $0.23 | $6.99 |
| Shelf Stable, Sunflower Seeds, Roasted & Salted, Original, Bag (*adj for yield*) | 163 | 30 | 1 | bag | $2.39 | $0.44 | $2.39 |
| Refrigerated, Pepperoni, Original, Pillow Pack, Resealable Zip-Pak | 170 | 10 | 1 | pkg | $5.15 | $0.30 | $5.15 |
| **SEASONINGS & MISCELLANEOUS** | | | | | | | |
| Fresh Parsley | 74 | 0.55 | 1 | bunch | $1.99 | $0.01 | $1.99 |
| Shelf Stable, Mustard, Original Yellow, Bottle | 340 | 8 | 1 | bottle | $2.29 | $0.05 | $2.29 |
| Shelf Stable, Seasoning, Black Pepper, Ground, Pure, Can | 85 | 1 | 1 | can | $5.99 | $0.07 | $5.99 |
| Shelf Stable, Seasoning, Garlic Powder, Jar | 88 | 1.1 | 1 | jar | $3.69 | $0.05 | $3.69 |
| Shelf Stable, Seasoning, Italian Seasoning, Dried, Jar | 21 | 0.09 | 1 | jar | $2.09 | $0.01 | $2.09 |
| Shelf Stable, Seasoning, Onion Powder, Jar | 74 | 1 | 1 | jar | $3.19 | $0.04 | $3.19 |
| Shelf Stable, Seasoning, Poultry Seasoning, Jar | 18 | 1.81 | 1 | jar | $3.79 | $0.38 | $3.79 |
| Shelf Stable, Seasoning, Taco Seasoning, Mild, Paleo, Preservative Free | 37 | 3 | 1 | pkg | $2.48 | $0.20 | $2.48 |
| **SWEETS/SNACK GROUP** | | | | | | | |
| Shelf Stable, Candy, Dark Chocolate, 70% Cocoa, Excellence, Bar | 100 | 50 | 1 | each | $3.39 | $1.70 | $3.39 |
| Shelf Stable, Candy, Mini Chips, 100% Real Chocolate, Semi-Sweet, Bag | 283 | 6.2 | 1 | bag | $7.05 | $0.15 | $7.05 |
| Shelf Stable, Chips, Tortilla, White Corn, Bag | 311.8 | 65 | 1 | bag | $2.69 | $0.56 | $2.69 |
| Shelf Stable, Cookies, Sandies, Cashew Shortbread | 317 | 60 | 1 | bag | $4.09 | $0.77 | $4.09 |
| Shelf Stable, Honey, Clover, US Grade A, No Drip Cap, Bottle | 340 | 60 | 1 | bottle | $4.39 | $0.77 | $4.39 |
| Shelf Stable, Cheetos, Crunchy, 8.5 oz Bag | 240 | 25 | 1 | bag | $5.69 | $0.59 | $5.69 |
| Shelf Stable, Syrup, Maple, 100% Pure, Bottle | 333 | 151.7 | 1 | bottle | $11.25 | $5.13 | $11.25 |
| **BREAD, CEREAL, GRAINS & PASTA GROUP** | | | | | | | |
| Shelf Stable, Cereal, Puffed Rice, Natural, Box | 170 | 4.7 | 1 | box | $2.99 | $0.08 | $2.99 |
| Shelf Stable, Corn Muffin, Mix, Box | 240 | 35.37 | 1 | bag | $0.89 | $0.13 | $0.89 |
| Shelf Stable, Egg Noodles, Extra Wide, Dry, Bag | 453 | 100 | 1 | bag | $3.29 | $0.73 | $3.29 |
| Shelf Stable, Macaroni, Elbow, Dry, Box | 453 | 140 | 1 | box | $1.79 | $0.55 | $1.79 |
| Shelf Stable, Macaroni, Rotini, Dry, Box | 453 | 90 | 1 | box | $1.79 | $0.36 | $1.79 |
| Shelf Stable, Cereal, Frosted Flakes, Dry, Box | 382 | 30 | 1 | box | $5.79 | $0.45 | $5.79 |
| Frozen, Pancakes, Buttermilk, Eggo, Box | 420 | 75 | 1 | box | $3.49 | $0.62 | $3.49 |
| Shelf Stable, Stuffing, Stove Top, Chicken, Box | 170 | 80 | 1 | box | $2.99 | $1.41 | $2.99 |
| **BAKERY/BAKED PRODUCT INGREDIENTS** | | | | | | | |
| Shelf Stable, Cocoa Powder, Unsweetened, Natural, Can | 226 | 13.44 | 1 | can | $5.09 | $0.30 | $5.09 |
| Shelf Stable, Extract, Almond, Pure, Bottle | 57 | 4.2 | 1 | bottle | $5.29 | $0.39 | $5.29 |
| Shelf Stable, Extract, Vanilla, Pure, Bottle | 29.4 | 10.5 | 1 | bottle | $4.89 | $1.75 | $4.89 |
| Shelf Stable, Flour, All Purpose, Enriched, Unbleached, Pre-sifted | 4530 | 379.49 | 1 | bag | $6.49 | $0.54 | $6.49 |
| Shelf Stable, Salt, Iodized, Table, Cylinder | 737 | 9.49 | 1 | each | $1.19 | $0.02 | $1.19 |
| Shelf Stable, Spice, Cinnamon, Ground, Jar | 67 | 1.41 | 1 | jar | $3.79 | $0.08 | $3.79 |
| Shelf Stable, Spice, Nutmeg, Ground, Jar | 31 | 0.09 | 1 | jar | $5.79 | $0.02 | $5.79 |
| Shelf Stable, Sugar, Light Brown, Bag | 907 | 27.5 | 1 | bag | $3.09 | $0.09 | $3.09 |
| Shelf Stable, Sugar, Powdered, Bag | 907 | 0.21 | 1 | bag | $3.59 | $0.00 | $3.59 |
| Shelf Stable, Sugar, White, Granulated, Beet Sugar | 1810 | 121.95 | 1 | bag | $3.09 | $0.21 | $3.09 |
| Shelf Stable, Yeast, Active Dry, 3-count Package | 21 | 1.69 | 1 | pkg | $1.89 | $0.15 | $1.89 |
| Shelf Stable, Yeast, Instant Dry, Quick Rise, Gluten Free, Jar | 113.4 | 7.28 | 1 | jar | $7.79 | $0.50 | $7.79 |

**TOTAL= $68.06 $378.87**

**$13.61 $75.77**

# Supplemental Table 11. Shelf Stability of Foods in More-Processed Western Menu

| **Food Name** | **Max. Expiration time (days)** | **Type of Storage before Expiration** | **Packaged Food? (Y/N)** | **INFO source** |
| --- | --- | --- | --- | --- |
| **DAY 1 BREAKFAST** | | | | |
| Cran-Rasp Juice Cocktail | 270 | shelf | Y | Greater Pittsburgh Community Food Bank: Shelf Life of Food Bank Products |
| Ham&Cheese Egg Bake | 4 | refrigerated | N | foodkeeper |
| Crispy Crowns | 365 | frozen | Y | foodkeeper |
| White Bread | 18 | shelf | Y | foodkeeper |
| Butter | 90 | refrigerated | Y | Greater Pittsburgh Community Food Bank: Shelf Life of Food Bank Products |
| Strawberry Jelly | 548 | refrigerated | Y | Greater Pittsburgh Community Food Bank: Shelf Life of Food Bank Products |
| **DAY 1 LUNCH** | | | | |
| Fruit Punch | 270 | shelf | Y | Greater Pittsburgh Community Food Bank: Shelf Life of Food Bank Products |
| Ground Beef | 2 | refrigerated | N | foodkeeper |
| Taco Seasoning | 730 | shelf | Y | Greater Pittsburgh Community Food Bank: Shelf Life of Food Bank Products |
| Iceberg Lettuce | 14 | refrigerated | N | foodkeeper |
| Grape Tomatoes | 7 | shelf | N | foodkeeper |
| Shred Cheddar | 30 | refrigerated | Y | foodkeeper |
| Sour Cream | 21 | refrigerated | Y | Greater Pittsburgh Community Food Bank: Shelf Life of Food Bank Products |
| Salsa | 548 | shelf | Y | Greater Pittsburgh Community Food Bank: Shelf Life of Food Bank Products |
| Tortilla Chips | 60 | shelf | Y | Greater Pittsburgh Community Food Bank: Shelf Life of Food Bank Products |
| Chocolate Oreos | 120 | shelf | Y | Greater Pittsburgh Community Food Bank: Shelf Life of Food Bank Products |
| **DAY 1 SUPPER** | | | | |
| Peach Punch | 270 | shelf | Y | Greater Pittsburgh Community Food Bank: Shelf Life of Food Bank Products |
| Stuffing | 365 | shelf | Y | Greater Pittsburgh Community Food Bank: Shelf Life of Food Bank Products |
| Sunflower Kernels | 365 | shelf | Y | foodkeeper |
| Chicken | 2 | refrigerated | N | foodkeeper |
| Chicken Gravy | 1095 | shelf | Y | foodkeeper |
| Green Beans | 5 | refrigerated | N | foodkeeper |
| White Dinner Roll | 18 | shelf | Y | foodkeeper |
| Butter | 90 | refrigerated | Y | Greater Pittsburgh Community Food Bank: Shelf Life of Food Bank Products |
| Brownie | 548 | shelf | Y | Greater Pittsburgh Community Food Bank: Shelf Life of Food Bank Products |
| **DAY 2 BREAKFAST** | | | | |
| Grape Juice Cocktail | 270 | shelf | Y | Greater Pittsburgh Community Food Bank: Shelf Life of Food Bank Products |
| Pancake | 90 | frozen | Y | Greater Pittsburgh Community Food Bank: Shelf Life of Food Bank Products |
| Butter | 90 | refrigerated | Y | Greater Pittsburgh Community Food Bank: Shelf Life of Food Bank Products |
| Syrup | 365 | shelf | Y | foodkeeper |
| Bacon | 7 | refrigerated | Y | FDA refrigerator and freezer |
| **DAY 2 LUNCH** | | | | |
| Lemonade | 270 | shelf | Y | Greater Pittsburgh Community Food Bank: Shelf Life of Food Bank Products |
| Chicken Burger | 4 | refrigerated | N | foodkeeper |
| White Hamburger Bun | 18 | shelf | Y | foodkeeper |
| Miracle Whip | 730 | shelf | Y | Greater Pittsburgh Community Food Bank: Shelf Life of Food Bank Products |
| Breaded Chicken Patty | 60 | frozen | Y | Greater Pittsburgh Community Food Bank: Shelf Life of Food Bank Products |
| Iceberg Lettuce | 14 | refrigerated | N | foodkeeper |
| Doritos | 60 | shelf | Y | Greater Pittsburgh Community Food Bank: Shelf Life of Food Bank Products |
| Cashews | 730 | shelf | Y | Greater Pittsburgh Community Food Bank: Shelf Life of Food Bank Products |
| Dark Chocolate | 548 | shelf | Y | Greater Pittsburgh Community Food Bank: Shelf Life of Food Bank Products |
| **DAY 2 SUPPER** | | | | |
| Chocolate 2% Milk | 7 | refrigerated | Y | Greater Pittsburgh Community Food Bank: Shelf Life of Food Bank Products |
| Hamburger Hotdish | 4 | refrigerated | N | foodkeeper |
| Elbow Macaroni | 1095 | shelf | Y | Greater Pittsburgh Community Food Bank: Shelf Life of Food Bank Products |
| Ground Beef | 2 | refrigerated | N | foodkeeper |
| Tomatoes w/ GPCO | 730 | shelf | Y | Greater Pittsburgh Community Food Bank: Shelf Life of Food Bank Products |
| Cond Tomato Soup | 730 | shelf | Y | Greater Pittsburgh Community Food Bank: Shelf Life of Food Bank Products |
| Corn | 365 | frozen | Y | Greater Pittsburgh Community Food Bank: Shelf Life of Food Bank Products |
| White Dinner Roll | 18 | shelf | Y | foodkeeper |
| Butter | 90 | refrigerated | Y | Greater Pittsburgh Community Food Bank: Shelf Life of Food Bank Products |
| **DAY 3 BREAKFAST** | | | | |
| Peach Punch | 270 | shelf | Y | Greater Pittsburgh Community Food Bank: Shelf Life of Food Bank Products |
| Scrambled Eggs | 4 | refrigerated | N | foodkeeper |
| Shred Cheddar | 180 | refrigerated | Y | foodkeeper |
| White Bread | 18 | shelf | Y | foodkeeper |
| Butter | 90 | refrigerated | Y | Greater Pittsburgh Community Food Bank: Shelf Life of Food Bank Products |
| Strawberry Jelly | 540 | shelf | Y | Greater Pittsburgh Community Food Bank: Shelf Life of Food Bank Products |
| **DAY 3 LUNCH** | | | | |
| Chocolate 2% Milk | 7 | refrigerated | Y | Greater Pittsburgh Community Food Bank: Shelf Life of Food Bank Products |
| Chili | 4 | refrigerated | N | foodkeeper |
| Chili Beans | 1095 | shelf | Y | Greater Pittsburgh Community Food Bank: Shelf Life of Food Bank Products |
| Ground Beef | 2 | refrigerated | N | foodkeeper |
| Celery | 14 | refrigerated | N | foodkeeper |
| Salsa | 540 | shelf | Y | Greater Pittsburgh Community Food Bank: Shelf Life of Food Bank Products |
| Sunflower Kernels | 365 | shelf | Y | foodkeeper |
| Cornbread mix | 270 | shelf | Y | foodkeeper |
| Honey | 730 | shelf | Y | Greater Pittsburgh Community Food Bank: Shelf Life of Food Bank Products |
| Cashew Shortbread | 120 | shelf | Y | Greater Pittsburgh Community Food Bank: Shelf Life of Food Bank Products |
| **DAY 3 SUPPER** | | | | |
| Fruit Punch | 270 | shelf | Y | Greater Pittsburgh Community Food Bank: Shelf Life of Food Bank Products |
| Pork & Noodles | 4 | refrigerated | N | foodkeeper |
| Egg Noodles | 1095 | shelf | Y | Greater Pittsburgh Community Food Bank: Shelf Life of Food Bank Products |
| Pork Loin | 5 | refrigerated | N | foodkeeper |
| Sour Cream | 21 | refrigerated | Y | Greater Pittsburgh Community Food Bank: Shelf Life of Food Bank Products |
| Beefy Mush Soup | 1095 | shelf | Y | Greater Pittsburgh Community Food Bank: Shelf Life of Food Bank Products |
| Peas | 365 | frozen | Y | Greater Pittsburgh Community Food Bank: Shelf Life of Food Bank Products |
| Steamed Petite Carrots | 28 | refrigerated | N | foodkeeper |
| Brownie | 548 | shelf | Y | Greater Pittsburgh Community Food Bank: Shelf Life of Food Bank Products |
| **DAY 4 BREAKFAST** | | | | |
| Cran-Rasp Juice Cocktail | 270 | shelf | Y | Greater Pittsburgh Community Food Bank: Shelf Life of Food Bank Products |
| Baked French Toast | 4 | refrigerated | N | foodkeeper |
| Custard Mix | 4 | refrigerated | N | foodkeeper |
| Texas Bread | 18 | shelf | Y | foodkeeper |
| Butter | 90 | refrigerated | Y | Greater Pittsburgh Community Food Bank: Shelf Life of Food Bank Products |
| Syrup | 365 | shelf | Y | foodkeeper |
| Bacon | 7 | refrigerated | Y | FDA refrigerator and freezer |
| **DAY 4 LUNCH** | | | | |
| Fruit Punch | 270 | shelf | Y | Greater Pittsburgh Community Food Bank: Shelf Life of Food Bank Products |
| Pizza Hotdish | 4 | refrigerated | N | foodkeeper |
| Spiral Macaroni | 1095 | shelf | Y | Greater Pittsburgh Community Food Bank: Shelf Life of Food Bank Products |
| Ground Beef | 2 | refrigerated | N | foodkeeper |
| Pepperoni | 30 | refrigerated | Y | Greater Pittsburgh Community Food Bank: Shelf Life of Food Bank Products |
| Pizza Sauce | 730 | shelf | Y | Greater Pittsburgh Community Food Bank: Shelf Life of Food Bank Products |
| Shred Mozzarella | 21 | refrigerated | Y | rouxbe refrigerator and freezer chart |
| Garlic Breadstick | 90 | frozen | Y | Greater Pittsburgh Community Food Bank: Shelf Life of Food Bank Products |
| Cashews | 730 | shelf | Y | Greater Pittsburgh Community Food Bank: Shelf Life of Food Bank Products |
| Chocolate Oreos | 120 | shelf | Y | Greater Pittsburgh Community Food Bank: Shelf Life of Food Bank Products |
| **DAY 4 SUPPER** | | | | |
| Chicken & Potatoes | 4 | refrigerated | N | foodkeeper |
| Red Potatoes | 60 | shelf | N | foodkeeper |
| Chicken | 2 | refrigerated | N | foodkeeper |
| Chicken Gravy | 1095 | shelf | Y | foodkeeper |
| Chicken Seasoning | 730 | shelf | Y | foodkeeper |
| Peas | 365 | frozen | Y | Greater Pittsburgh Community Food Bank: Shelf Life of Food Bank Products |
| White Dinner Roll | 18 | shelf | Y | Greater Pittsburgh Community Food Bank: Shelf Life of Food Bank Products |
| Butter | 90 | shelf | Y | Greater Pittsburgh Community Food Bank: Shelf Life of Food Bank Products |
| Rice Krispie Bar | 365 | shelf | Y | foodkeeper |
| **DAY 5 BREAKFAST** | | | | |
| Grape Juice Cocktail | 270 | shelf | Y | Greater Pittsburgh Community Food Bank: Shelf Life of Food Bank Products |
| 1% Milk | 7 | refrigerated | Y | foodkeeper |
| Frosted Corn Flakes | 365 | shelf | Y | foodkeeper |
| White Bread | 18 | shelf | Y | foodkeeper |
| Sunflower Butter | 548 | shelf | Y | Greater Pittsburgh Community Food Bank: Shelf Life of Food Bank Products |
| Honey | 730 | shelf | Y | Greater Pittsburgh Community Food Bank: Shelf Life of Food Bank Products |
| **DAY 5 LUNCH** | | | | |
| Lemonade | 270 | shelf | Y | Greater Pittsburgh Community Food Bank: Shelf Life of Food Bank Products |
| Ham Sandwich | 5 | refrigerated | N | foodkeeper |
| White Hamburger Bun | 18 | shelf | Y | Greater Pittsburgh Community Food Bank: Shelf Life of Food Bank Products |
| Mustard | 730 | refrigerated | Y | Shelf life chart of pantry foods (featured video) |
| Sliced Ham | 14 | refrigerated | Y | foodkeeper |
| Iceberg Lettuce | 14 | refrigerated | N | foodkeeper |
| Macaroni Salad | 5 | refrigerated | N | foodkeeper |
| Elbow Macaroni | 1095 | shelf | Y | Greater Pittsburgh Community Food Bank: Shelf Life of Food Bank Products |
| Petite Carrots | 28 | refrigerated | N | foodkeeper |
| Celery | 14 | refrigerated | N | foodkeeper |
| Peas | 365 | frozen | Y | Greater Pittsburgh Community Food Bank: Shelf Life of Food Bank Products |
| Green Onions | 7 | refrigerated | N | foodkeeper |
| Miracle Whip | 730 | shelf | Y | Greater Pittsburgh Community Food Bank: Shelf Life of Food Bank Products |
| Mustard | 730 | shelf | Y | Shelf life chart of pantry foods (featured video) |
| Cheetos | 60 | shelf | Y | Greater Pittsburgh Community Food Bank: Shelf Life of Food Bank Products |
| Dark Chocolate | 548 | shelf | Y | Greater Pittsburgh Community Food Bank: Shelf Life of Food Bank Products |
| **DAY 5 SUPPER** | | | | |
| Chocolate 2% Milk | 7 | refrigerated | Y | Greater Pittsburgh Community Food Bank: Shelf Life of Food Bank Products |
| Tator Tot Hotdish | 4 | refrigerated | N | foodkeeper |
| Ground Beef | 2 | refrigerated | N | foodkeeper |
| Corn | 365 | frozen | Y | Greater Pittsburgh Community Food Bank: Shelf Life of Food Bank Products |
| Cond Mushr Soup | 1095 | shelf | Y | Greater Pittsburgh Community Food Bank: Shelf Life of Food Bank Products |
| Onion Powder | 1095 | shelf | Y | Shelf life chart of pantry foods (featured video) |
| Garlic Powder | 1095 | shelf | Y | Shelf life chart of pantry foods (featured video) |
| Crispy Crowns* | 365 | frozen | Y | foodkeeper |
| White Dinner Roll | 18 | shelf | Y | foodkeeper |
| Butter | 90 | refrigerated | Y | Greater Pittsburgh Community Food Bank: Shelf Life of Food Bank Products |
| Cashew Shortbread | 120 | shelf | Y | Greater Pittsburgh Community Food Bank: Shelf Life of Food Bank Products |

Supplemental Table 12. Shelf Stability of Foods in Less-Processed Western Menu

| **Food Name** | **Max. Expiration time (days)** | **Type of Storage before Expiration** | **Packaged Food? (Y/N)** | **INFO source** |
| --- | --- | --- | --- | --- |
| **Day 1 Breakfast** | | | | |
| Cranberry Juice | 270 | shelf | Y | Greater Pittsburgh Community Food Bank: Shelf Life of Food Bank Products |
| Prosciutto Egg Cups | 4 | refrigerated | N | foodkeeper |
| Prosciutto | 14 | refrigerated | Y | Greater Pittsburgh Community Food Bank: Shelf Life of Food Bank Products |
| Eggs | 35 | refrigerated | N | foodkeeper |
| Homemade White Bread | 5 | shelf | N | foodkeeper |
| Butter | 90 | refrigerated | Y | Greater Pittsburgh Community Food Bank: Shelf Life of Food Bank Products |
| Berry Jam without Pectin | 21 | refrigerated | N | foodkeeper |
| **DAY 1 LUNCH** | | | | |
| Fruit Punch | 6 | refrigerated | N | foodkeeper |
| Taco Meat | 2 | refrigerated | N | foodkeeper |
| Ground Beef | 2 | refrigerated | N | foodkeeper |
| Taco Seasoning | 730 | shelf | Y | Greater Pittsburg Community Food Bank: Shelf Life of Food Bank Products |
| Iceberg Lettuce | 14 | refrigerated | N | foodkeeper |
| Grape Tomatoes | 7 | shelf | N | foodkeeper |
| Shredded Pepperjack | 30 | refrigerated | Y | foodkeeper |
| Sour Cream | 21 | refrigerated | Y | Greater Pittsburgh Community Food Bank: Shelf Life of Food Bank Products |
| Salsa | 548 | shelf | Y | Greater Pittsburgh Community Food Bank: Shelf Life of Food Bank Products |
| White Corn Tortilla Chips | 60 | shelf | Y | Greater Pittsburgh Community Food Bank: Shelf Life of Food Bank Products |
| **DAY 1 SUPPER** | | | | |
| Lemonade | 6 | refrigerated | N | foodkeeper |
| Chicken & Stuffing | 4 | refrigerated | N | foodkeeper |
| Butter | 90 | refrigerated | Y | Greater Pittsburgh Community Food Bank: Shelf Life of Food Bank Products |
| Sunflower Seeds | 365 | shelf | Y | Greater Pittsburgh Community Food Bank: Shelf Life of Food Bank Products |
| Chicken | 2 | refrigerated | N | foodkeeper |
| Gravy | 1095 | shelf | Y | foodkeeper |
| Green Beans | 5 | refrigerated | N | foodkeeper |
| Dinner Rolls from Scratch | 5 | shelf | N | foodkeeper |
| Butter | 90 | refrigerated | Y | Greater Pittsburgh Community Food Bank: Shelf Life of Food Bank Products |
| Homemade Brownies | 5 | shelf | N | foodkeeper |
| **DAY 2 BREAKFAST** | | | | |
| Grape Juice | 270 | shelf | Y | Greater Pittsburg Community Food Bank: Shelf Life of Food Bank Products |
| Pancake Mix | 60 | refrigerated | Y | Greater Pittsburg Community Food Bank: Shelf Life of Food Bank Products |
| Butter | 90 | refrigerated | Y | Greater Pittsburg Community Food Bank: Shelf Life of Food Bank Products |
| Maple Syrup | 365 | shelf | Y | foodkeeper |
| Bacon | 7 | refrigerated | Y | FDA Refrigerator and Freezer |
| **DAY 2 LUNCH** | | | | |
| Lemonade | 6 | refrigerated | N | foodkeeper |
| Chicken Sandwich | 4 | refrigerated | N | foodkeeper |
| Chicken Strips | 60 | frozen | Y | Greater Pittsburg Community Food Bank: Shelf Life of Food Bank Products |
| Homemade Hamburger Buns | 5 | shelf | N | foodkeeper |
| Mayo | 730 | refrigerated | Y | Greater Pittsburg Community Food Bank: Shelf Life of Food Bank Products |
| Iceberg Lettuce | 14 | refrigerated | N | foodkeeper |
| White Corn Tortilla Chips | 60 | shelf | Y | Greater Pittsburg Community Food Bank: Shelf Life of Food Bank Products |
| Cashews | 730 | shelf | Y | Greater Pittsburg Community Food Bank: Shelf Life of Food Bank Products |
| Milk Chocolate | 548 | shelf | Y | Greater Pittsburg Community Food Bank: Shelf Life of Food Bank Products |
| **DAY 2 SUPPER** | | | | |
| Pasteurized Milk | 7 | refrigerated | Y | foodkeeper |
| Hamburger Hotdish | 4 | refrigerated | N | foodkeeper |
| Elbow Macaroni | 1095 | shelf | Y | Greater |
| Ground Beef | 2 | refrigerated | N | foodkeeper |
| Regular Tomatoes | 7 | shelf | N | foodkeeper |
| Canned Soup | 730 | shelf | Y | Greater Pittsburg Community Food Bank: Shelf Life of Food Bank Products |
| Corn | 365 | frozen | Y | Greater Pittsburg Community Food Bank: Shelf Life of Food Bank Products |
| Dinner Rolls from Scratch | 5 | shelf | N | foodkeeper |
| Butter | 90 | refrigerated | Y | Greater Pittsburgh Community Food Bank: Shelf Life of Food Bank Products |
| Homemade Brownies | 5 | shelf | N | foodkeeper |
| **DAY 3 BREAKFAST** | | | | |
| Vanilla Steamer | 5 | refrigerated | N | foodkeeper |
| Scrambled Eggs | 4 | refrigerated | N | foodkeeper |
| Shredded Chedder | 180 | refrigerated | Y | Greater Pittsburgh Community Food Bank: Shelf Life of Food Bank Products |
| Homemade White Bread | 5 | shelf | N | foodkeeper |
| Butter | 90 | refrigerated | Y | Greater Pittsburgh Community Food Bank: Shelf Life of Food Bank Products |
| Jam without Pectin | 21 | refrigerated | N | foodkeeper |
| **DAY 3 LUNCH** | | | | |
| Pasteurized Milk | 7 | refrigerated | Y | foodkeeper |
| Slow Cooker Beans | 4 | refrigerated | N | foodkeeper |
| Ground Beef | 2 | refrigerated | N | foodkeeper |
| Celery | 14 | refrigerated | N | foodkeeper |
| Sunflower Seeds | 365 | shelf | Y | Greater Pittsburgh Community Food Bank: Shelf Life of Food Bank Products |
| Cornbread mix | 270 | shelf | Y | foodkeeper |
| Honey | 730 | shelf | Y | Greater Pittsburgh Community Food Bank: Shelf Life of Food Bank Products |
| Wedding Cookies | 120 | shelf | Y | Greater Pittsburgh Community Food Bank: Shelf Life of Food Bank Products |
| **DAY 3 SUPPER** | | | | |
| Fruit Punch | 6 | refrigerated | N | foodkeeper |
| Pork & Noodles | 4 | refrigerated | N | foodkeeper |
| Egg Noodles | 1095 | shelf | Y | Greater Pittsburgh Community Food Bank: Shelf Life of Food Bank Products |
| Pork Tenderloin | 5 | refrigerated | N | foodkeeper |
| Sour Cream | 5 | refrigerated | Y | Greater Pittsburgh Community Food Bank: Shelf Life of Food Bank Products |
| Mushroom Soup | 1095 | shelf | Y | Greater Pittsburgh Community Food Bank: Shelf Life of Food Bank Products |
| Peas (frozen) | 365 | frozen | Y | Greater Pittsburgh Community Food Bank: Shelf Life of Food Bank Products |
| Steamed Petite Carrots | 28 | refrigerated | N | foodkeeper |
| Homemade Brownies | 5 | shelf | N | foodkeeper |
| **DAY 4 BREAKFAST** | | | | |
| Apple Juice | 270 | shelf | Y | Greater Pittsburgh Community Food Bank: Shelf Life of Food Bank Products |
| Ricotta Stuffed French Toast | 4 | refrigerated | N | foodkeeper |
| Butter | 90 | refrigerated | Y | Greater Pittsburgh Community Food Bank: Shelf Life of Food Bank Products |
| Maple Syrup | 365 | shelf | Y | Greater Pittsburgh Community Food Bank: Shelf Life of Food Bank Products |
| Bacon | 7 | refrigerated | Y | FDA Refrigerator and Freezer |
| **DAY 4 LUNCH** | | | | |
| Fruit Punch | 6 | refrigerated | N | foodkeeper |
| Pizza Hotdish | 4 | refrigerated | N | foodkeeper |
| Spiral Macaroni | 1095 | shelf | Y | Greater Pittsburgh Community Food Bank: Shelf Life of Food Bank Products |
| Ground Beef | 2 | refrigerated | N | foodkeeper |
| Pepperoni | 30 | refrigerated | Y | Greater Pittsburgh Community Food Bank: Shelf Life of Food Bank Products |
| Pizza Sauce | 730 | shelf | Y | Greater Pittsburgh Community Food Bank: Shelf Life of Food Bank Products |
| Shredded Mozzarella | 21 | refrigerated | Y | rouxbe refrigerator and freezer chart |
| Homemade Breadsticks | 5 | shelf | N | foodkeeper |
| Cashews | 730 | shelf | Y | Greater Pittsburgh Community Food Bank: Shelf Life of Food Bank Products |
| Wedding Cookies | 120 | shelf | Y | Greater Pittsburgh Community Food Bank: Shelf Life of Food Bank Products |
| **DAY 4 SUPPER** | | | | |
| Chicken & Potatoes | 4 | refrigerated | N | foodkeeper |
| Red Potatoes | 60 | shelf | N | foodkeeper |
| Chicken | 2 | refrigerated | N | foodkeeper |
| Gravy | 1095 | shelf | Y | Greater Pittsburgh Community Food Bank: Shelf Life of Food Bank Products |
| Chicken Seasoning | 730 | shelf | Y | foodkeeper |
| Peas | 365 | frozen | Y | Greater Pittsburgh Community Food Bank: Shelf Life of Food Bank Products |
| Homemade Dinner Rolls | 5 | shelf | N | foodkeeper |
| Butter | 90 | refrigerated | Y | Greater Pittsburgh Community Food Bank: Shelf Life of Food Bank Products |
| Homemade Krispie Treats | 5 | shelf | N | foodkeeper |
| **DAY 5 BREAKFAST** | | | | |
| Grape Juice | 270 | shelf | Y | Greater Pittsburgh Community Food Bank: Shelf Life of Food Bank Products |
| Almond Steamer | 5 | refrigerated | N | foodkeeper |
| Simple Syrup | 14 | refrigerated | N | foodkeeper |
| Instant Oatmeal | 365 | shelf | Y | Greater Pittsburgh Community Food Bank: Shelf Life of Food Bank Products |
| Homemade White Bread | 5 | shelf | N | foodkeeper |
| Sunflower Butter | 548 | shelf | Y | Greater Pittsburgh Community Food Bank: Shelf Life of Food Bank Products |
| **DAY 5 LUNCH** | | | | |
| Lemonade | 6 | refrigerated | N | foodkeeper |
| Ham Sandwich | 5 | refrigerated | N | foodkeeper |
| Homemade Hamburger Buns | 5 | shelf | N | foodkeeper |
| Yellow Mustard | 730 | shelf | Y | Shelf Life of Pantry foods (featured video) |
| Deli Ham | 14 | refrigerated | Y | foodkeeper |
| Iceberg Lettuce | 14 | refrigerated | N | foodkeeper |
| Macaroni Salad | 5 | refrigerated | N | foodkeeper |
| Elbow Macaroni | 1095 | shelf | Y | Greater Pittsburgh Community Food Bank: Shelf Life of Food Bank Products |
| Petite Carrots | 28 | refrigerated | N | foodkeeper |
| Celery | 14 | refrigerated | N | foodkeeper |
| Peas | 365 | frozen | Y | Greater Pittsburgh Community Food Bank: Shelf Life of Food Bank Products |
| Green Onions | 7 | refrigerated | N | foodkeeper |
| Mayo | 730 | shelf | Y | Greater Pittsburgh Community Food Bank: Shelf Life of Food Bank Products |
| Mustard | 730 | shelf | Y | Shelf Life of Pantry foods (featured video) |
| Paleo Puffs | 90 | shelf | Y | Greater Pittsburgh Community Food Bank: Shelf Life of Food Bank Products |
| Chocolate | 548 | shelf | Y | Greater Pittsburgh Community Food Bank: Shelf Life of Food Bank Products |
| **DAY 5 SUPPER** | | | | |
| Pasteurized Milk | 7 | refrigerated | Y | foodkeeper |
| Tater Tot Hotdish | 4 | refrigerated | N | foodkeeper |
| Ground Beef | 2 | refrigerated | N | foodkeeper |
| Corn | 365 | frozen | Y | Greater Pittsburgh Community Food Bank: Shelf Life of Food Bank Products |
| Mushroom Soup | 1095 | shelf | Y | Greater Pittsburgh Community Food Bank: Shelf Life of Food Bank Products |
| Onion Powder | 1095 | shelf | Y | Shelf life chart of pantry foods (featured video) |
| Garlic Powder | 1095 | shelf | Y | Shelf life chart of pantry foods (featured video) |
| Homemade Tater Tots | 4 | refrigerated | N | foodkeeper |
| Homemade Dinner Rolls | 5 | shelf | N | foodkeeper |
| Butter | 90 | refrigerated | Y | Greater Pittsburgh Community Food Bank: Shelf Life of Food Bank Products |
| Wedding Cookies | 120 | shelf | Y | Greater Pittsburgh Community Food Bank: Shelf Life of Food Bank Products |

1. Items changed from original Less-Processed Western Menu [↑](#footnote-ref-1)
