# Supplementary material for: Using Less Processed Food to Mimic a Standard American Diet Does Not Improve Nutrient Value and May Result in a Shorter Shelf Life at a Higher Financial Cost
Source: Curr Dev Nutr. 2024 Oct 2;8(11):104471. doi: 10.1016/j.cdnut.2024.104471 (PMC11539364; doi:10.1016/j.cdnut.2024.104471)
Supplement: Supplementary file 2 — Multimedia component 2 [file mmc2.pptx]

## Slide 1
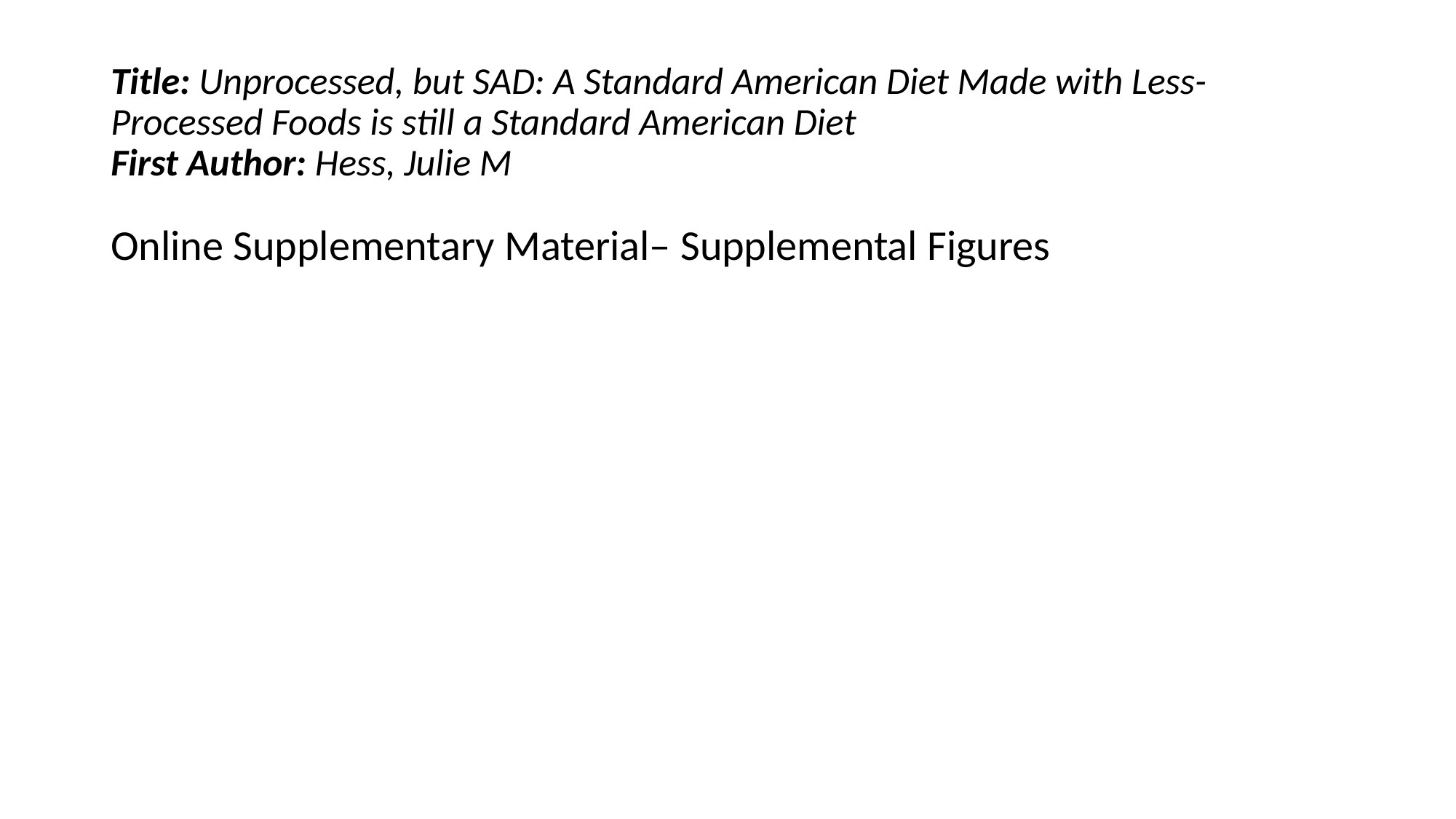

# Title: Unprocessed, but SAD: A Standard American Diet Made with Less-Processed Foods is still a Standard American Diet First Author: Hess, Julie M
Online Supplementary Material– Supplemental Figures

## Slide 2
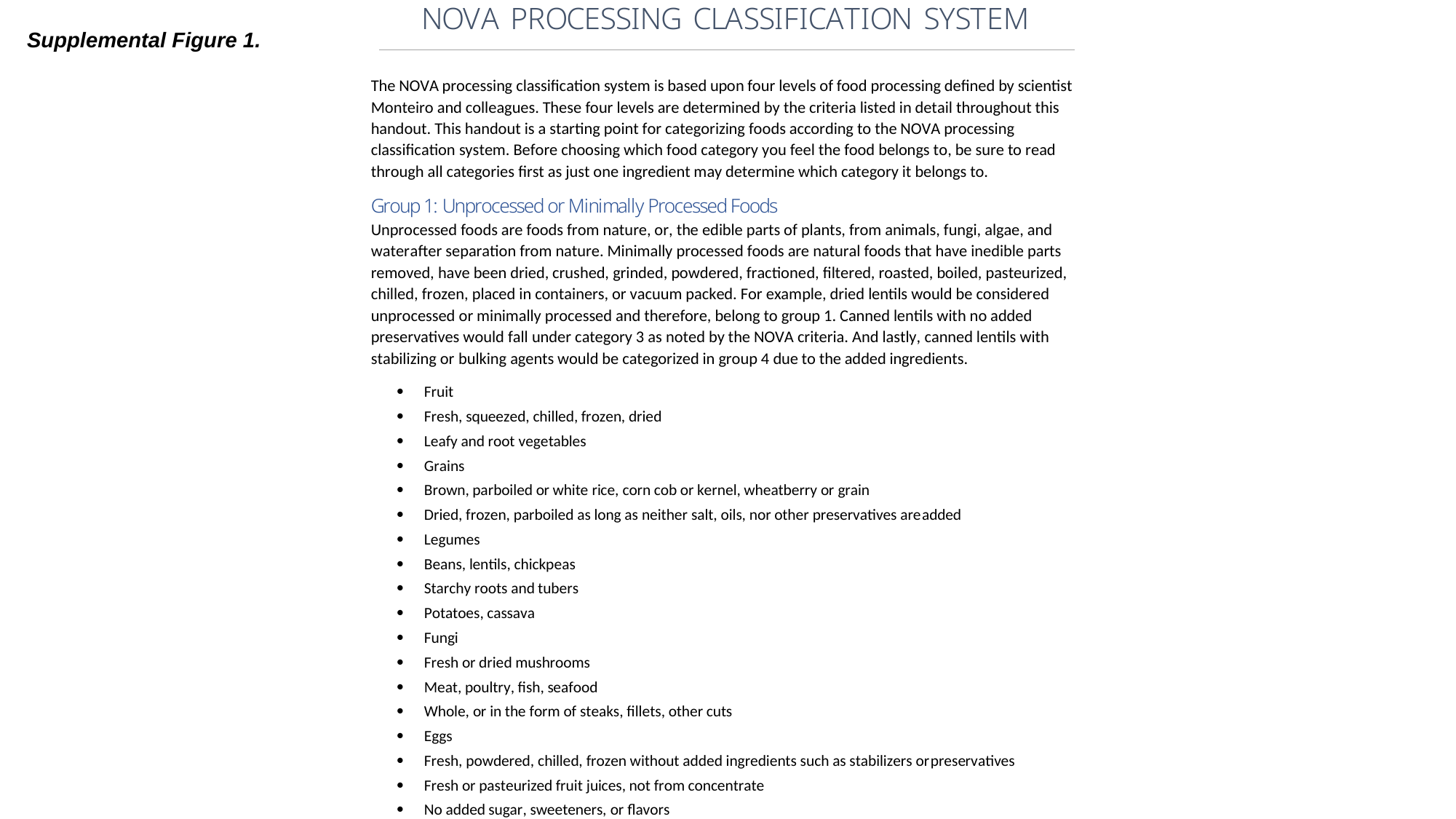

Supplemental Figure 1.

## Slide 3
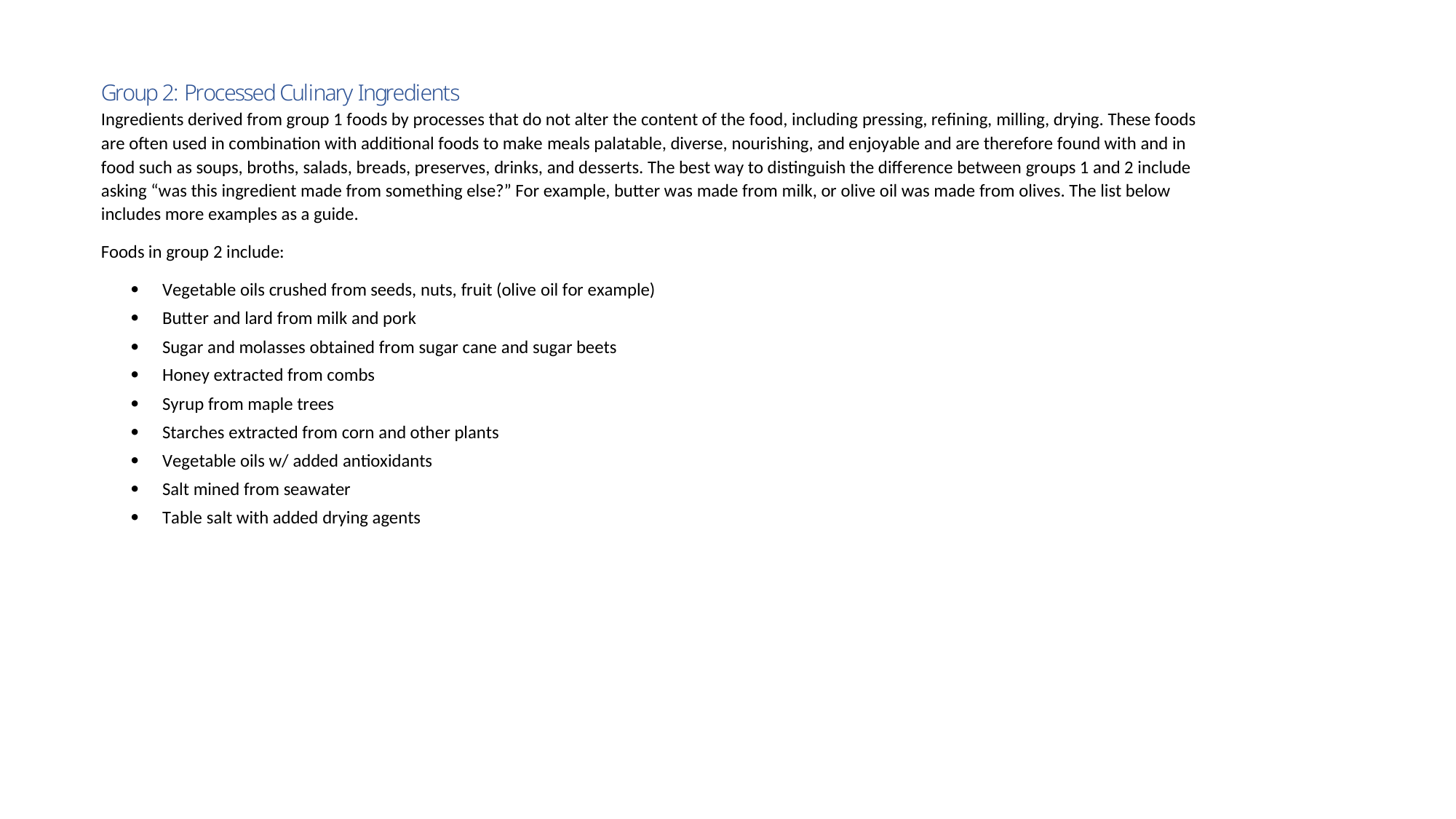

## Slide 4
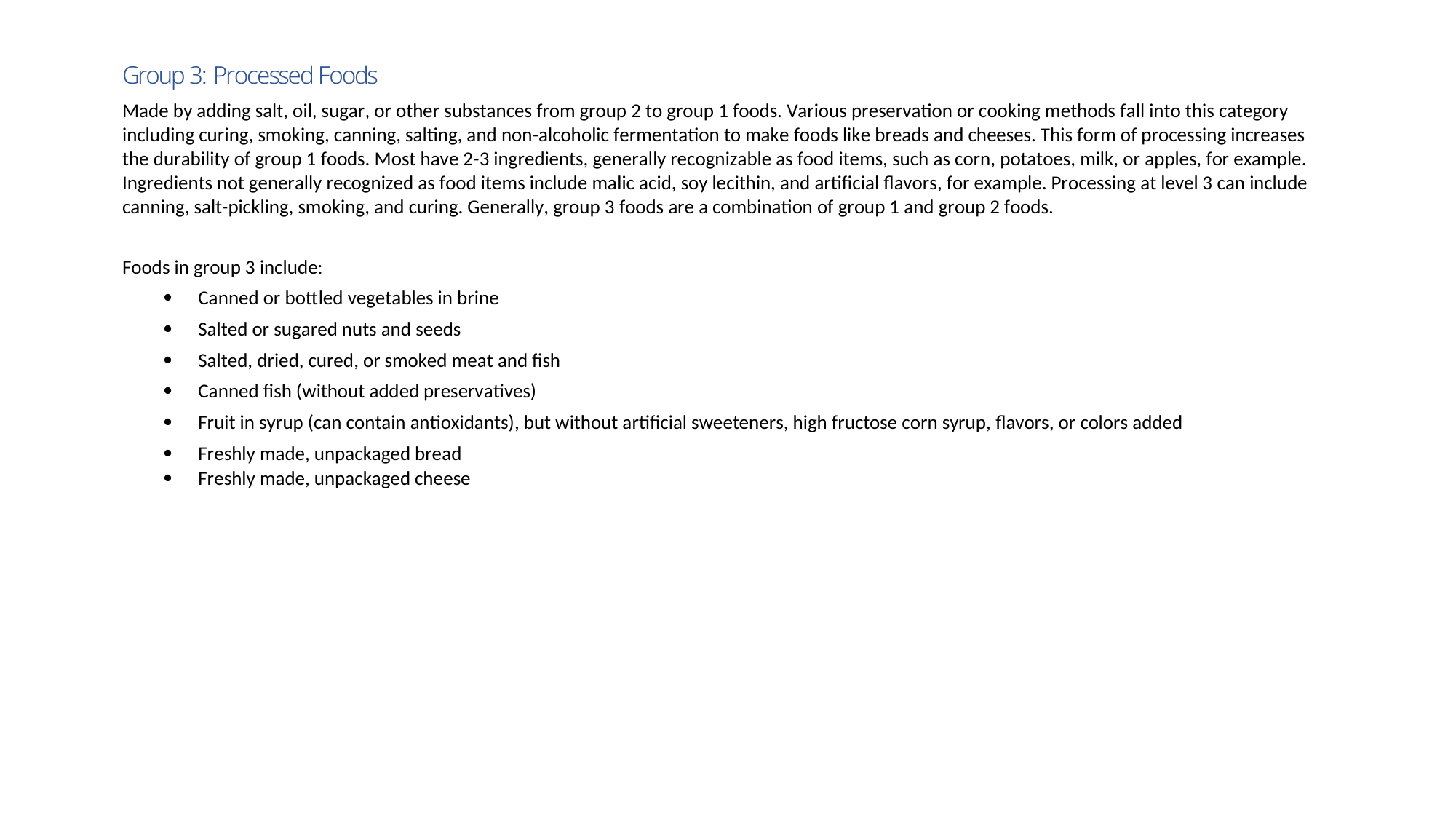

## Slide 5
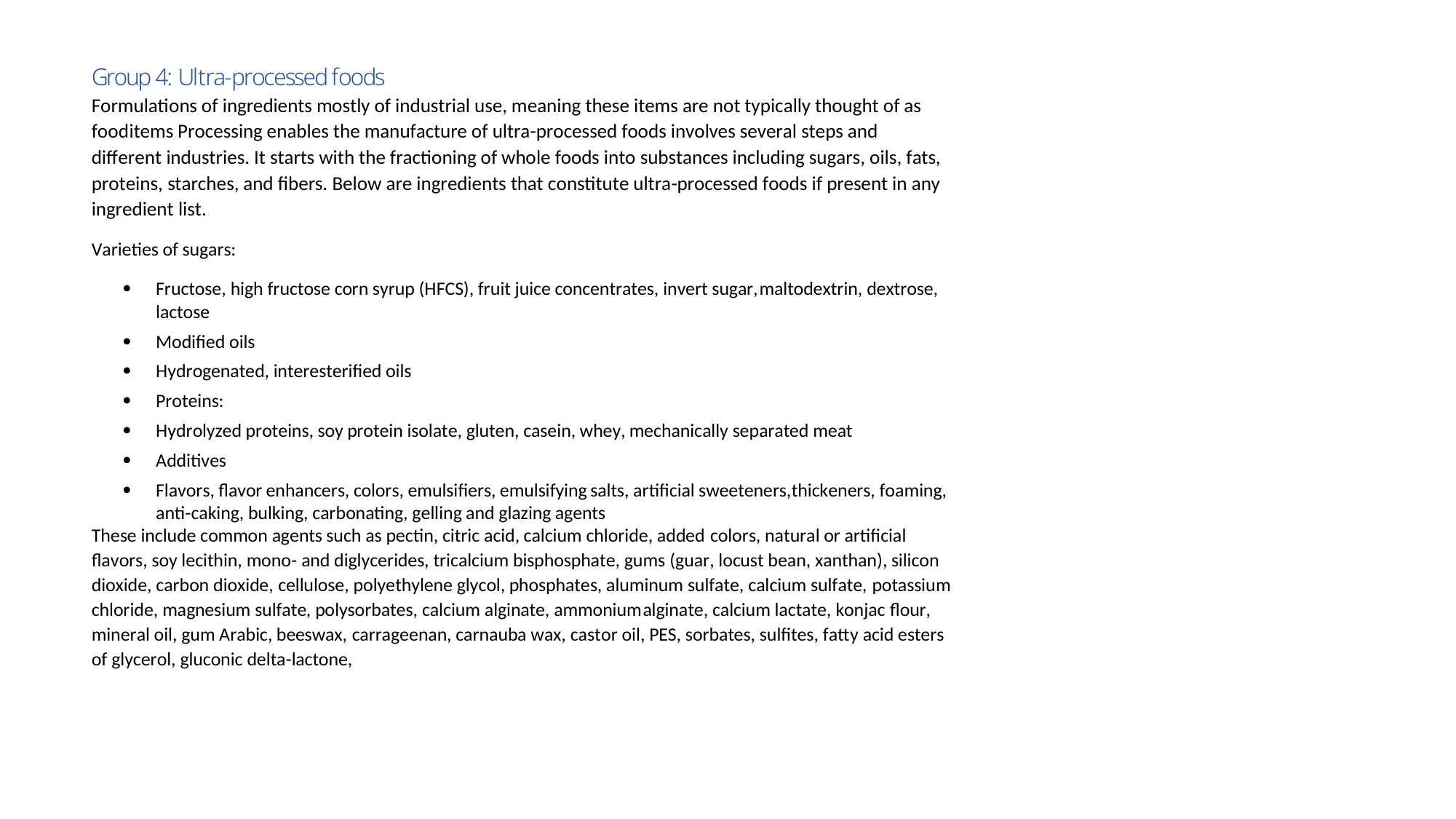

## Slide 6
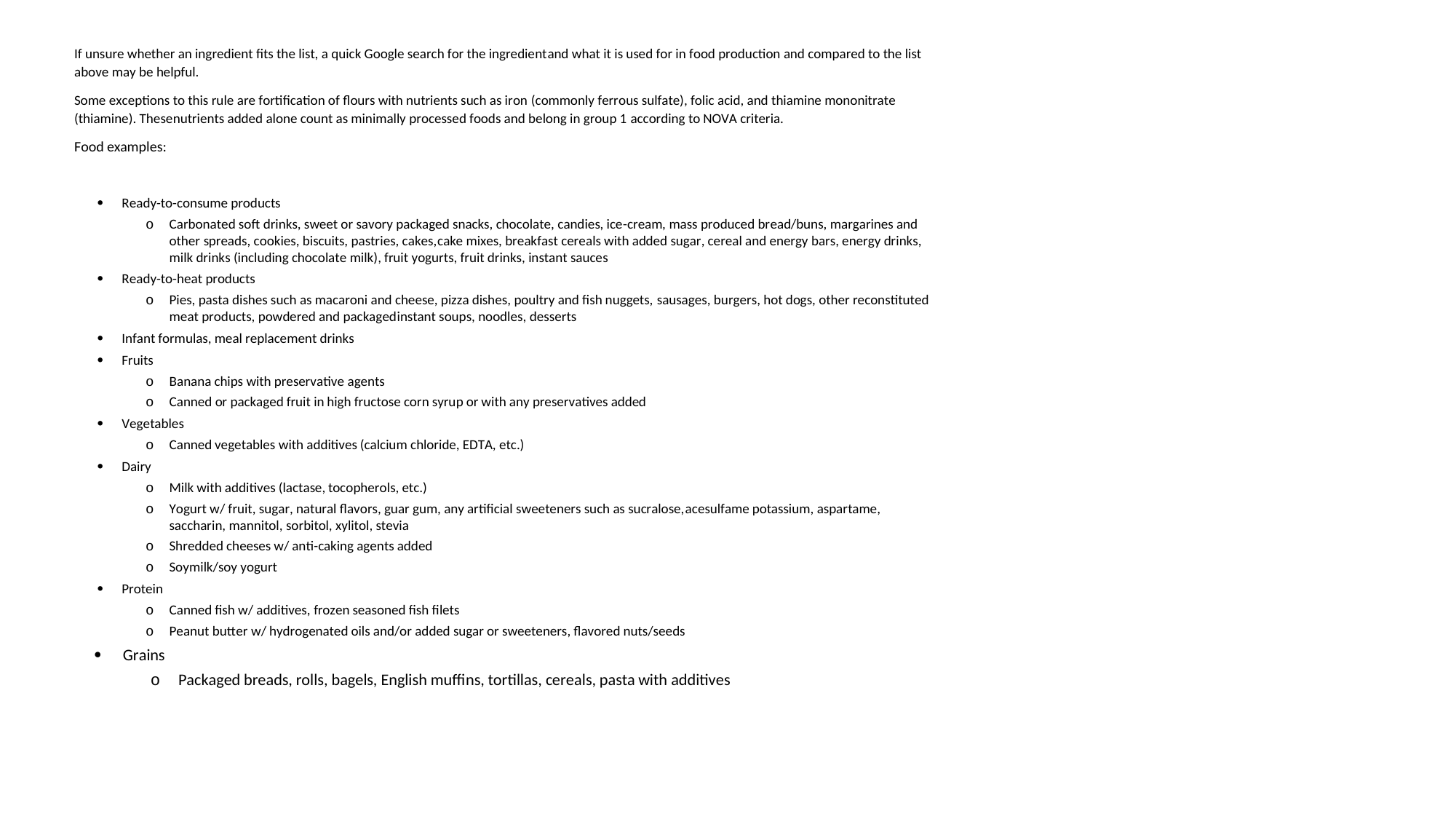

## Slide 7
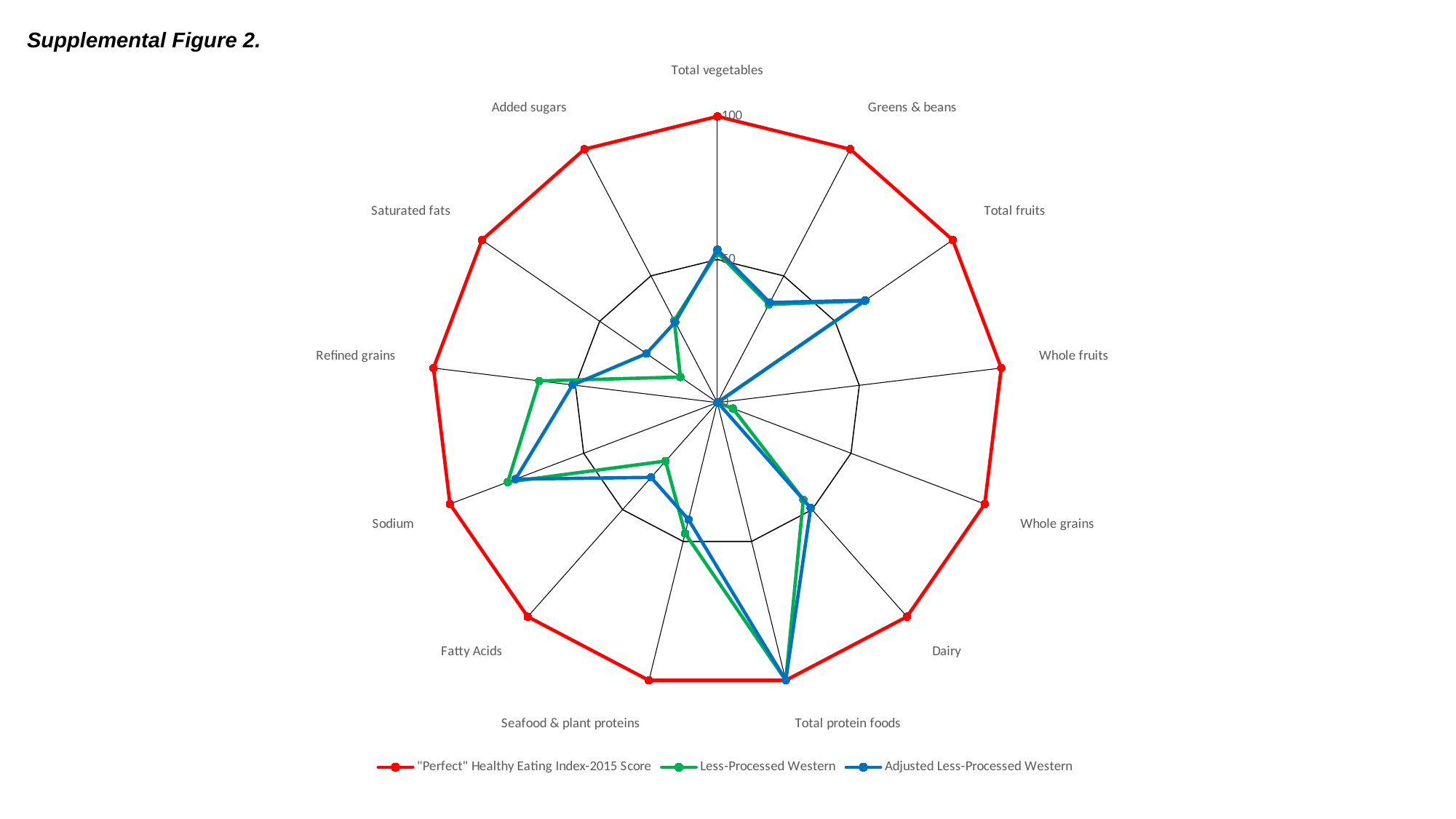

Supplemental Figure 2.
### Chart
| Category | "Perfect" Healthy Eating Index-2015 Score | Less-Processed Western | Adjusted Less-Processed Western |
|---|---|---|---|
| Total vegetables | 100.0 | 52.407579999999996 | 53.438418 |
| Greens & beans | 100.0 | 38.700511999999996 | 39.469698 |
| Total fruits | 100.0 | 62.56721400000001 | 62.885723999999996 |
| Whole fruits | 100.0 | 0.0 | 0.0 |
| Whole grains | 100.0 | 5.773694 | 0.23047600000000004 |
| Dairy | 100.0 | 45.339633 | 49.224934 |
| Total protein foods | 100.0 | 100.0 | 100.0 |
| Seafood & plant proteins | 100.0 | 47.093697 | 42.12951699999999 |
| Fatty Acids | 100.0 | 27.333360000000003 | 34.955892 |
| Sodium | 100.0 | 78.385554 | 75.473558 |
| Refined grains | 100.0 | 62.76486 | 50.995248000000004 |
| Saturated fats | 100.0 | 15.705552 | 30.125725000000003 |
| Added sugars | 100.0 | 32.394267 | 31.767113999999996 |
